# Rapid amyloid-β clearance and cognitive recovery through multivalent modulation of blood–brain barrier transport

**DOI:** 10.1038/s41392-025-02426-1

**Published:** 2025-10-07

**Authors:** Junyang Chen, Pan Xiang, Aroa Duro-Castano, Huawei Cai, Bin Guo, Xiqin Liu, Yifan Yu, Su Lui, Kui Luo, Bowen Ke, Lorena Ruiz-Pérez, Qiyong Gong, Xiaohe Tian, Giuseppe Battaglia

**Affiliations:** 1 Department of Radiology, Huaxi MR Research Center (HMRRC), Institute of Radiology and Medical Imaging, West China Hospital of Sichuan University, Chengdu, Sichuan China; 2 Xiamen Key Lab of Psychoradiology and Neuromodulation, Department of Radiology, West China Xiamen Hospital of Sichuan University, Xiamen, Fujian China; 3 Institute for Bioengineering of Catalunya (IBEC), The Barcelona Institute of Science and Technology, Barcelona, Spain; 4 Department of Chemistry and Institute for Physics of Living Systems, University College London (UCL), London, UK; 5 Laboratory of Aging Research and Cancer Drug Target, State Key Laboratory of Biotherapy and Cancer Center, National Clinical Research Center for Geriatrics, West China Hospital, Sichuan University, Chengdu, Sichuan,, China; 6 Curapath, Paterna, Valencia Spain; 7 Serra Hunter Fellow, Department of Applied Physics, University of Barcelona, Barcelona, Spain; 8 Research Unit of Psychoradiology, Chinese Academy of Medical Sciences, Chengdu, Sichuan China; 9 Catalan Institution for Research and Advanced Studies (ICREA), Barcelona, Spain

**Keywords:** Neurological disorders, Blood-brain barrier, Biophysics, Drug development, Nanobiotechnology

## Abstract

The blood‒brain barrier (BBB) is a highly selective permeability barrier that safeguards the central nervous system (CNS) from potentially harmful substances while regulating the transport of essential molecules. Its dysfunction is increasingly recognized as a pivotal factor in the pathogenesis of Alzheimer’s disease (AD), contributing to the accumulation of amyloid-β (Aβ) plaques. We present a novel therapeutic strategy that targets low-density lipoprotein receptor-related protein 1 (LRP1) on the BBB. Our design leverages the multivalent nature and precise size of LRP1-targeted polymersomes to modulate receptor-mediated transport, biasing LRP1 trafficking toward transcytosis and thereby upregulating its expression to promote efficient Aβ removal. In AD model mice, this intervention significantly reduced brain Aβ levels by nearly 45% and increased plasma Aβ levels by 8-fold within 2 h, as measured by ELISA. Multiple imaging techniques confirmed the reduction in brain Aβ signals after treatment. Cognitive assessments revealed that treated AD mice exhibited significant improvements in spatial learning and memory, with performance levels comparable to those of wild-type mice. These cognitive benefits persisted for up to 6 months post-treatment. This work pioneers a new paradigm in drug design, where function arises from the supramolecular nature of the nanomedicine, harnessing multivalency to elicit biological action at the membrane trafficking level. Our findings also reaffirm the critical role of the BBB in AD pathogenesis and demonstrate that targeting the BBB can make therapeutic interventions significantly more effective. We establish a compelling case for BBB modulation and LRP1-mediated Aβ clearance as a transformative foundation for future AD therapies.

## Introduction

Alzheimer’s disease (AD) accounts for almost 70% of dementia cases, and its pathophysiology is characterized by an accumulation of small peptides, amyloid-β (Aβ), in fibrils and plaques, followed by hyperphosphorylation, misfolding, and aggregation into neurofibrillary tangles of another protein, tau. Both aggregates are associated with strong inflammatory responses, synaptic dysfunction, and neuronal injury, causing considerable brain damage and impairing cognitive processes.^1,2^ In addition, the brain vasculature network, often referred to as the blood-brain barrier (BBB), plays a critical role in AD progression and possibly initiation.^3–6^ The BBB consists of aligned endothelial cells supported by pericytes and astrocytes, forming the densest vascular network in the body, with approximately one capillary per neuron.^3^ Mounting evidence indicates that BBB dysfunction actively drives AD pathogenesis through interconnected pathological cascades: perivascular Aβ deposition progressively accumulates,^7–10^ while low-density lipoprotein receptor-related protein 1 (LRP1) localization shifts from endothelial cells to pericytes, a cell-specific redistribution significantly impairs Aβ clearance capacity and promotes neurovascular uncoupling pathogenesis.^11–13^ The BBB poses a significant challenge in pharmacology, impeding the penetration of most known drugs and complicating the discovery of treatments for neurological disorders.^3^ Most AD patients experience various vascular dysfunctions,^14^ which may be linked to Aβ^15^ and tau^16–18^ or occur independently of both.^19^ The LRP1 is possibly the most studied receptor for both Aβ^1,20–22^ and, more recently, tau^23,24^ processing. Endothelial LRP1 plays a vital role in removing Aβ, and its expression decreases with age. This decrease is more pronounced in AD patients and animal models, where BBB LRP1 levels are almost undetectable.^25–29^ The downregulation of LRP1 is strongly correlated with impairment of the BBB and cognitive decline.^27,30–34^ Proper regulation of LRP1 levels in endothelial cells is crucial for preventing the progression of AD. Despite this, the mechanisms that maintain appropriate LRP1 levels on the basolateral surface of endothelial cells remain unclear.

**Fig. 1:**
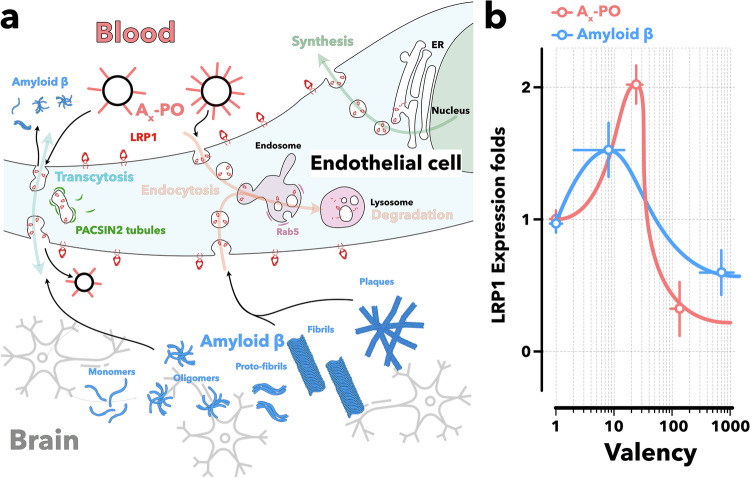
Schematics of LRP1 shuttling across brain endothelial cells (**a**) following either the PACSIN2 or Rab5 pathway and its relationship with multivalent cargo. LPR1 expression in brain endothelial cells (**b**) as a function of cargo valency. The latter represents the average number of ligands per cargo that interact with LRP1, with *n* = 1 representing a single peptide. The data are replotted from refs. ^31,32^

LRP1-mediated trafficking follows distinct routes influenced by the avidity of the cargo–receptor interaction. High-avidity binding promotes receptor clustering and recruitment of phosphatidylinositol-binding clathrin assembly protein (PICALM), triggering clathrin-mediated endocytosis and Rab5-dependent sorting within early endosomes. This pathway frequently leads to lysosomal degradation, thereby reducing the pool of membrane-bound LRP1 available for further transport. Mid-avidity cargo instead engages PACSIN2 (also known as syndapin-2), an F-BAR membrane-sculpting protein that generates and stabilizes tubular carriers linking the luminal and abluminal membranes. This noncanonical transcytosis pathway bypasses the degradative endo-lysosomal system, enabling rapid, degradation-free delivery of LRP1 cargo to the brain parenchyma or, in the case of efflux, into the circulation. For Aβ, the choice of pathway is crucial. Aβ/LRP1 complexes internalized through PICALM-clathrin-Rab5 processing may be recycled via Rab11-mediated transcytosis into the bloodstream^30–37^ or diverted into Rab7-positive compartments for lysosomal degradation.^38,39^ Our previous work^40,41^ revealed an alternative, Rab5/PICALM-independent route in which LRP1 is internalized collectively, trafficked in PACSIN2-stabilized tubular carriers, and exocytosed across the BBB (Fig. 1a). These two mechanisms have opposing functional outcomes: PACSIN2-mediated tubular trafficking preserves Aβ clearance capacity, whereas Rab5-dependent lysosomal routing reduces LRP1 surface availability and can accelerate amyloidogenic aggregation. Binding energetics underlie this sorting decision—mid-avidity interactions favor PACSIN2-mediated cycling, while high avidity triggers Rab5-directed degradation.^1,3,40–42^

Cargo avidity also influences LRP1 expression levels (Fig. 1b). Mid-avidity ligands promote PACSIN2-dependent shuttling, which correlates with sustained or even elevated LRP1 expression, likely because receptors are spared from degradation. In contrast, high-avidity ligands bias trafficking toward Rab5-rich endosomes and lysosomal processing, leading to LRP1 downregulation. We propose that LRP1 homeostasis reflects a dynamic balance between receptor synthesis and degradation, with a fraction of LRP1 normally cycling across the BBB via PACSIN2 carriers. New cargo can disrupt this equilibrium; high avidity shifts the balance toward degradation, lowering receptor levels, while mid avidity supports efficient shuttling and receptor preservation. Mid-affinity targeting is not a new idea in BBB drug delivery.^43–47^ Several neuropharmaceutical strategies already harness moderate binding strength to the transferrin receptor to bypass lysosomal degradation and improve central nervous system (CNS) uptake. These approaches have opened an important avenue in neurotherapeutics by exploiting binding energetics to enhance transport efficiency. However, they share a common limitation: they treat the BBB merely as a gate to cross rather than as a dysfunctional tissue to repair. In AD, the problem extends beyond access; the very transport machinery itself is pathologically biased. In the case of LRP1, we and others showed that it is increasingly sequestered in Rab5-positive endosomes by high-avidity Aβ aggregates, leading to receptor degradation and impaired clearance capacity. Our recent findings reframe avidity not only as a means of transport optimization but as a therapeutic switch capable of correcting faulty receptor trafficking. In healthy conditions, a fraction of LRP1 continuously cycles via PACSIN2-stabilized tubular carriers, preserving receptor levels and supporting Aβ efflux. In disease, high-avidity Aβ disrupts this balance, diverting receptors into degradative compartments and accelerating surface LRP1 depletion. By contrast, mid-avidity ligands bias trafficking toward PACSIN2-dependent tubular transcytosis, sparing receptors from degradation and promoting receptor upregulation (Fig. 1b). This creates an opportunity to reprogram LRP1 trafficking to restore BBB clearance function and reverse vascular dysfunction at its source. Guided by this concept, we developed angiopep-2–conjugated LRP1-targeted polymersomes (A_40_-POs) engineered for intermediate binding affinity. This energetically tuned design avoids the two pathological extremes: (i) high-affinity interactions that trigger Rab5-dependent lysosomal sequestration, and (ii) low-affinity dissociations that fail to sustain transport. Instead, A_40_-POs stabilize the LRP1–PACSIN2 transportosome, restoring endogenous tubular transcytosis and preserving receptor homeostasis. Unlike transferrin receptor–based mid-affinity systems, which aim only to boost cargo penetration, our platform actively repairs the endothelial trafficking defect, rebalancing LRP1 synthesis and degradation dynamics.

We propose that this “avidity-optimized trafficking reprogramming” approach can counteract Aβ-induced BBB dysfunction. Our superselective design—multivalent angiopep-2 on P[(OEG)_10_MA]_20_–PDPA_120_ polymersomes—targets LRP1 with mid-avidity to bias trafficking toward PACSIN2-mediated transcytosis, thereby promoting LRP1 upregulation and enhancing Aβ clearance.^40–42,48^ In preclinical testing, A_40_-POs achieved triple therapeutic synergy: rapid clearance of 41% of brain Aβ within hours via reactivated transcytosis; structural BBB restoration with 78% recovery of LRP1–CD31 colocalization; and long-lasting cognitive rescue, with Morris water maze performance indistinguishable from wild-type controls for 6 months. This performance surpasses antibody-based approaches, which are hampered by delayed onset, transient efficacy, and receptor depletion.^1,3,49^ By directly leveraging avidity as a therapeutic variable, our work extends mid-affinity targeting beyond simple delivery toward vascular repair, offering a universal and disease-modifying framework for neurological disorders in which BBB dysfunction and receptor downregulation are both cause and consequence of pathology.

## Results

**Fig. 2:**
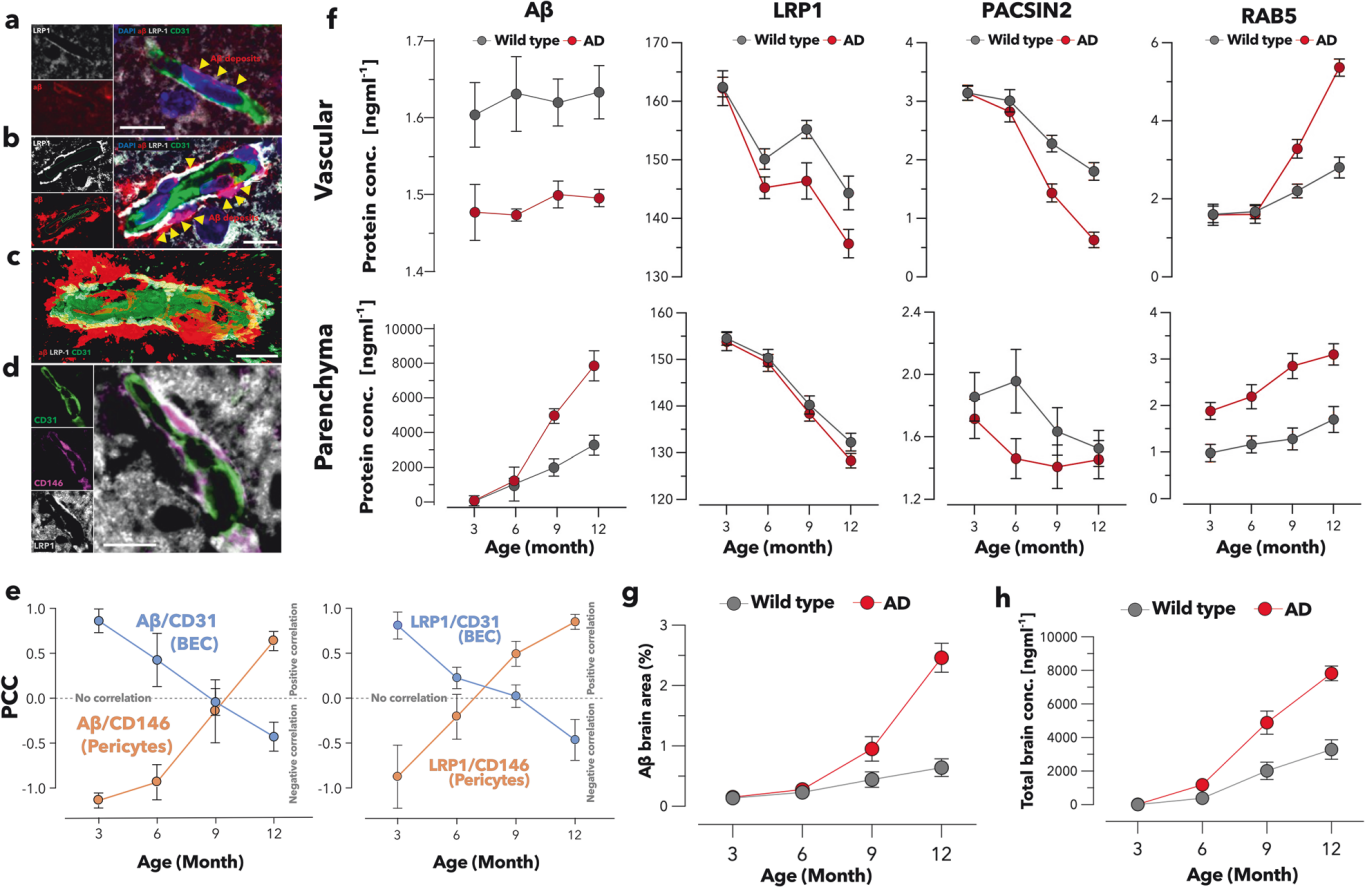
Comparative analysis of protein expression profiles in AD and wild-type mice. Confocal microscopy of brain endothelial cells, pericytes, and Aβ in AD mice of different ages. LRP1 (gray), Aβ (red), and BBB endothelium (CD31, green) in 3-month-old (**a**) and 12-month-old (**b**) AD mice brain samples. 3D rendering (**c**) of the 12-month-old AD brain section highlighting the spatial relationship between the different markers. In later AD stages, increased Aβ accumulation near the BBB and reduced colocalization of CD31 with LRP1 are observed. CD31, CD146, and LRP1 expression in a 12-month-old AD brain sample (**d**). Scale bar = 10 μm. Age-dependent colocalization quantification between LRP1/Aβ and endothelial cells (CD31) as well as between LRP1/Aβ and pericytes (CD146) in AD mice using PCC (**e**). Studise was performed on images derived from three independent experiments, with 6–7 vessels analysis per trial, the data are presented as the means ± SEMs. Quantification of Aβ, LRP1, PACSIN2, and Rab5 levels as a function of age by ELISA of the brain vasculature and parenchyma (**f**) in AD and wild-type mice. Quantitative representation of Aβ-positive area fraction in brain coronal sections identified through immunohistochemistry (IHC) in both AD model and wild-type mice (**g**). Aβ concentrations via ELISA in both AD and wild-type mice in the whole brain (**h**). (For graphs **f**, **g**, **h**, *n* ≥ 3 per group, the data are presented as the means ± SEMs.)

To fully understand the role of BBB LPR1 in mediating Aβ transport, we followed its expression alongside other markers and Aβ in the APP/PS1 and wild-type animals. We conducted a multi-tiered comparative investigation employing enzyme-linked immunosorbent assay (ELISA) quantification and in situ imaging modalities. This integrated methodology facilitated comprehensive whole-brain profiling coupled with systematic evaluation of separated vascular-parenchymal (Fig. 2 and supplementary Fig. 1).

We used confocal microscopy to assess the spatial localization of LRP1 and Aβ at the BBB endothelial cells (CD31) and pericytes (CD146) in 3- and 12-month-old AD brain samples. In Fig. 2a (3-month-old), Aβ (red) is highly colocalized with LRP1 (white) on the endothelial cells (green), suggesting the active involvement of LRP1 in Aβ transport and clearance at a younger age, with less Aβ accumulation around the vessels. Figure 2b shows 12-month-old brains, and the corresponding 3D image (Fig. 2c) indicates a noticeable increase in Aβ deposition on the basal side of the BBB vessels. The colocalization of LRP1 with Aβ appears to decrease, potentially indicating impaired LRP1-mediated clearance of Aβ as AD progresses. In the later stages of AD, increased Aβ accumulation and reduced association with LRP1 may affect BBB function and promote disease pathology. Interestingly, further imaging (Fig. 2d) suggested that LRP1 was predominantly deposited around the pericytes on the exterior side of the blood vessels. We conducted longitudinal analysis of representative brain sections spanning 3 to 12 months through immunolabeling with antibodies targeting LRP1, Aβ, pericyte marker (CD146), and endothelial cell marker (CD31). Colocalization analysis was performed, and Pearson correlation coefficients (PCC) were calculated to quantify the spatial relationships between Aβ/LRP1 and CD31, as well as between Aβ/LRP1 and CD146. In Fig. 2e, the results show a trend where the association between Aβ and endothelial cells weakens over time, while its correlation with pericytes appears to strengthen. Similarly, analyses with LRP1 revealed PCC over time, specifically between LRP1 and endothelial cells as well as between LRP1 and pericytes, suggesting potential associations.

We collected brains from both AD and wild-type mice over a lifespan of 3–12 months and fractionated them into parenchyma and vasculature. We thus measured Aβ, LRP1, PACSIN2, and Rab5 levels via ELISA. The data shown in Fig. 2f reveal significantly more Aβ in the vasculature of wild-type mice with notable differences emerging at all lifespan stages compared with APP/PS1 mice, demonstrating the pathological hallmarks of AD. These differences correspond to increased Aβ levels in the parenchyma. The latter is the dominant index of temporal changes in Aβ from the macroscopic whole brain. Figure 2g (quantitative data of supplementary Fig. 1) and Fig. 2h display the aggregate Aβ levels in the brain, showing a marked Aβ increase in the AD models with age, a trend particularly pronounced between 6 and 12 months. The buildup of Aβ in brain, along with its restricted passage through blood vessels, corresponds to the downregulation of LRP1 and PACSIN2, alongside the upregulation of Rab5 as the animals aged, as measured by ELISA (Fig. 2f), and immune fluorescence (supplementary Fig. 2). Notably, this difference between AD and wild-type animals was especially significant during the 6- to 12-month period, particularly in the vascular system (Fig. 2f and supplementary Fig. 2). The interplay between LRP1, PACSIN2, and Rab5 at the BBB is crucial for understanding the mechanisms of aging and AD. Our previous studies^40,42,50^ in which the peptide angiopep-2 was used to target LRP1 revealed that the efficiency of crossing the BBB is greater for multivalent scaffolds. We demonstrated that LRP1 shuttles across the BBB through transcytosis^31^ via collective endocytosis and exocytosis regulated by the BAR domain protein PACSIN2 for mid-avidity cargo. PACSIN2 plays a pivotal role in facilitating transport via LRP1 for small Aβ structures (i.e., mid-avidity cargo) across the BBB.^41^ A large Aβ structure with greater affinity for LRP1 traffics toward Rab5-positive endosomes via the recruitment of PICALM and clathrin-mediated endocytosis.^35^ The loss of BBB integrity may trigger compensatory mechanisms, including the upregulation of Rab5, as the brain attempts to increase endosomal trafficking to manage increased cellular stress and the accumulation of neurotoxic substances, such as Aβ. However, Rab5 is significantly upregulated in vulnerable neuronal populations, particularly in individuals with AD.^51,52^ Combining the above ELISA and confocal evaluation results, the localization shift in LRP1 from the BBB vascular endothelium to pericytes with aging underscores a potentially pivotal role in the pathophysiology of AD. This progression suggests a decrease in LRP1-mediated Aβ clearance at the BBB endothelial level, with a concomitant increase associated with pericytes, which may impact AD progression. Most importantly, the timing of this alteration precedes or evolves alongside the early stage of cognitive decline, as measured in the APP/PS1 AD model we used.^50^

As we previously reported, both small Aβ and mid-avidity multivalent units trigger PACSIN2-mediated transcytosis. In both, this pathway is associated with the upregulation of the LRP1 receptor,^31,32^ as shown in Fig. 1b. We thus hypothesize that the use of multivalent LRP1-targeted nanoparticles may restore the ability of LRP1 to transport Aβ from the brain and potentially clear Aβ deposits in AD models. We prepared and characterized P[(OEG)_10_ MA]_20_-PDPA_120_ mixed with angiopep2-P[(OEG)_10_ MA]_20_-PDPA_120_ to make polymersomes bearing 40 ligands per particle. Hereinafter, these polymersomes are referred to as A_40_-POs (supplementary Fig. 3). The number of ligands optimized for transcytosis^31^ was adjusted via our phenotypic targeting theory calculations to account for the reduced LRP1 expression in AD mice.^34^

**Fig. 3:**
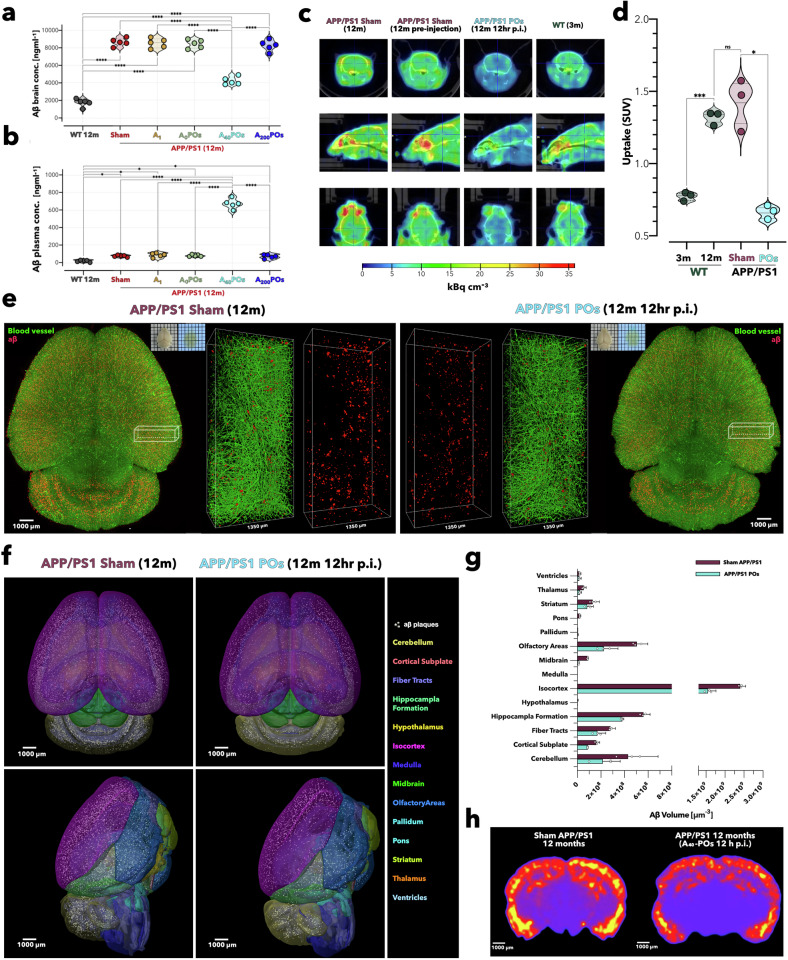
A_40_-POs treatment reduces cerebral Aβ burden in APP/PS1 mice. ELISA measurement of whole-brain (**a**) and plasma (**b**) Aβ levels at 2 h post injection, comparing wild-type (WT), sham, angiopep-2 alone (A_1_), pristine P[(OEG)_10_ MA]_20_-PDPA_120_ polymersomes (A_0_-POs), and angiopep-2-functionalized polymersomes with ligand densities of 40 (A_40_-POs) or 200 (A_200_-POs) per vesicle. PET‒CT visualization (**c**) in wild-type (WT) and APP/PS1 (pre- and 12 h post A_40_-POs administration) mice with [^18^ F] AV-45 (2.8--3.2 MBq) tracer. Quantified SUV (**d**) reductions confirm significant Aβ clearance. 3D rendering imaging (**e**) of the brain after tissue clearing shows reduced Aβ signals in 12 h post A_40_-POs injected mice. Brain parcellation into 14 regions was performed according to the Allen Brain Atlas, revealing Aβ distribution across brain regions (**f**). A_40_-POs treatment induced 41% Aβ volume reduction in total after 12 h of treatment (**g**) (data expressed as mean ± SEM). Heat map representation of Aβ fluorescence intensity across a single coronal brain layer (**h**). Tissue-clearing and PET-CT imaging were conducted three repeats per group (*n* = 3). Statistical significance for graphs **a**, **b**, **d** was determined via one-way analysis of variance (ANOVA), *p < 0.05, **p < 0.01, ***p < 0.001, ****p < 0.0001

APP/PS1 transgenic AD mice were intravenously injected with 200 μL of A_40_-POs alongside four control treatments: a sham formulation (only PBS), angiopep-2 alone (A1), pristine P[(OEG)_10_ MA]_20_-PDPA_120_ polymersomes (A_0_-POs), and polymersomes with 200 angiopep-2 ligands (A_200_-POs). Two hours after administration, the animals were culled, and the Aβ levels in both the brain and blood plasma were measured via ELISA. The results plotted in Fig. 3a, b show a clear effect on only A_40_-POs treatment, with a reduction in brain Aβ of almost 50%, from 8603.6 to 4236.3 ng ml^−1^, and a mirrored increase in the blood plasma of 8 times from 85.3 to 673.5 ng ml^−1^ compared with that of the diseased animals treated with a sham formulation. If we assume that the brain volume of an APP/PS1 12-month-old mouse brain is 0.35–0.45 ml and has a total blood volume of 1.5–2.3 ml, the amount of Aβ removed from the brain corresponds almost entirely to the surplus measured in the plasma.

A parallel IHC analysis confirmed that the Aβ area fraction also decreased (supplementary Fig. 4). Furthermore, we employed positron emission tomography-computed tomography (PET-CT) to assess the clearance of Aβ in the brains of live animals. The animals were injected with [^18 ^F]-4-(2-(6-(2-(2-(2-18F-fluoroethoxy)ethoxy)ethoxy)pyridin-3-yl)vinyl)-N-methyl benzamine ([^18 ^F] AV-45), an established Aβ marker.^53^ PET‒CT revealed that the brain of 12-month-old APP/PS1 mice exhibited intense Aβ signal. In contrast, this signal sharply decreased after treatment with A_40_-POs (Fig. 3c). After 12 h of administration of A_40_-POs, the reduction in [^18 ^F] AV-45 standardized uptake value associated with Aβ was 46.25% (Fig. 3d). Confocal images revealed that Aβ deposition around the BBB disappeared, and a large amount of Aβ signal in the vascular lumen (supplementary Fig. 5). We performed tissue clearing on the brains of 12-month-old APP/PS1 mice treated with sham formulation or A_40_-POs. The Aβ (red) and blood vessels (green) of these brains were labeled (Fig. 3e and supplementary Fig. 6). The brains of the mice treated with A_40_-POs presented fewer Aβ signals than did the Sham APP/PS1 brains. The 3D brain images were embedded into the Allen Brain Atlas-based parcellation model integrated with Amira software, with each brain parcellated into 14 distinct regions (Fig. 3f). The Aβ volume in 14 brain regions of the mouse brain was measured separately (Fig. 3g). There was a 41% Aβ volume reduction in the brains of A_40_-POs-treated mice. Finally, the coronal Aβ distribution is shown as a heatmap in Fig. 3h.

**Fig. 4:**
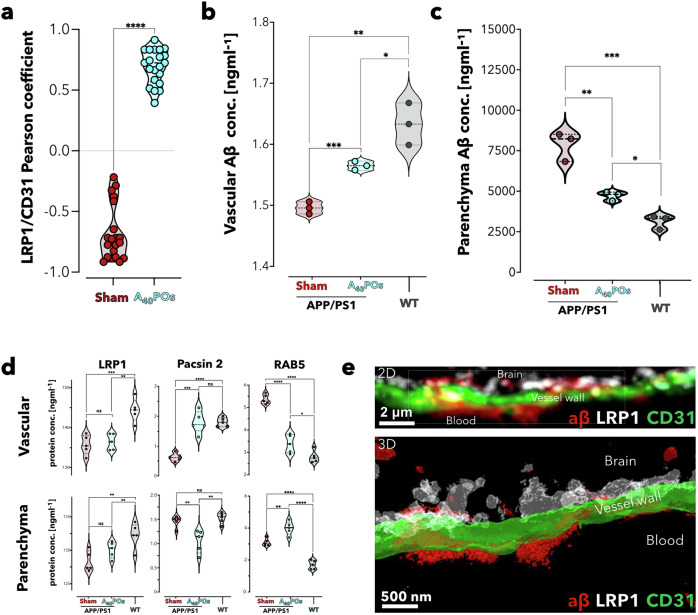
A_40_-POs treatment restored the BBB phenotype. PCC for the colocalization of LRP1 and endothelial cells (CD31) (**a**). Analyzis was performed on images derived from three independent experiments, with 6–7 vessels studied per trial (statistical analysis performed via unpaired t-tests, **** *p* < 0.0001). Aβ content in cerebrovasculature (**b**) and parenchyma (**c**) quantified by ELISA. ELISA measurements of vascular and parenchymal LRP1, PACSIN2, and Rab5 levels in wild-type, Sham APP/PS1, and APP/PS1 mice after A_40_-PO treatment (**d**). STED microscopy imaging of LRP1 (white), Aβ (red), and vessel wall (green). After treatment, Aβ deposits around the BBB are cleared, and notable Aβ signals are present within the vascular lumen (**e**). For **b**, **c**, and **d**, statistical significance was determined via one-way ANOVA, *p < 0.05, **p < 0.01, ***p < 0.001, ****p < 0.0001, *n* ≥ 3

These findings motivated us to study the BBB vascular phenotype after A_40_-PO treatment. We first observed increasing of the colocalization of LRP1 with CD31 in the treated brain, as shown in Fig. 4a and supplementary Fig. 7. The overlap of LRP1 and BBB endothelial cells (CD31) returned to the wild-type state. Quantitative analysis of Aβ distribution revealed a significant increase in the brain vasculature after treatment (Fig. 4b), contrasting with a progressive reduction in parenchymal Aβ deposition (Fig. 4c). ELISA tests were subsequently performed to detect proteins in both the vasculature and parenchyma. As discussed previously, the analysis focused on the concentrations of various proteins, including LRP1, PACSIN2, and Rab5. The nanomedicine cleared Aβ and caused a rapid change in the BBB phenotype by upregulating PACSIN2 and downregulating Rab5 (Fig. 4d). This finding is consistent with our fluorescent imaging data, which show that PACSIN2 relocates to blood vessels (supplementary Fig. 7). The morphology of LRP1, as observed under a stimulated emission depletion (STED) microscope, revealed a clustered distribution in the vessel wall, suggesting robust ongoing transcytosis (Fig. 4e and supplementary Fig. 5).

**Fig. 5:**
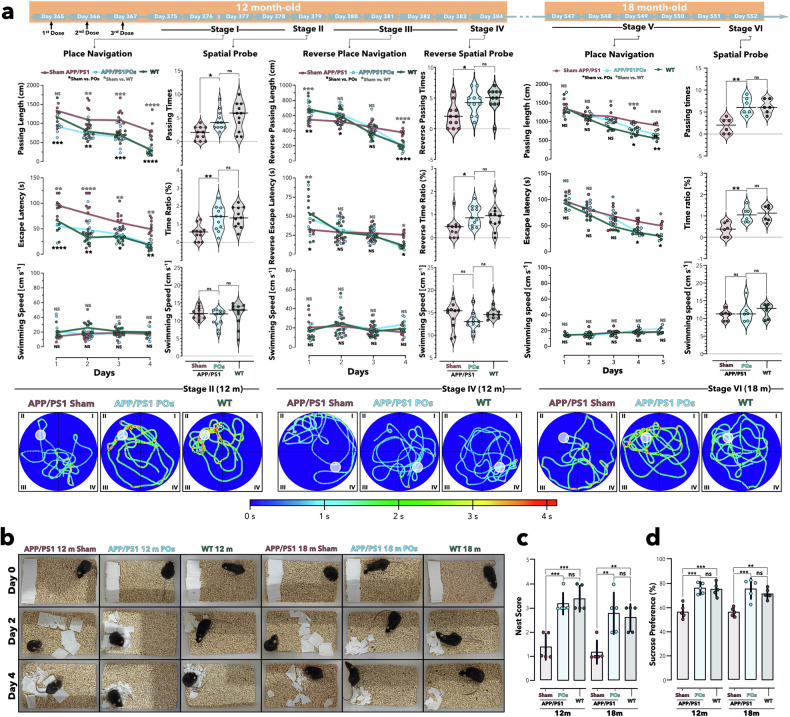
Behavior tests demonstrated that A_40_-PO treatment improved the performance of APP/PS1 mice. Morris water maze test in which mice were injected with saline (sham APP/PS1 group and WT group, 200 μL) or A_40_-POs (APP/PS1 POs group, 10 g/L 200 μL) once daily for the 365th–367th morning of their lifespan. Recovery was executed for 1 week under the original rearing conditions. The place navigation test (Stage I) was performed on days 375th–378th, and the results revealed a gradual decrease in passing length and escape latency for finding the platform in all groups, with the APP/PS1 POs group matching the WT group and significantly outperforming the Sham APP/PS1 group. During the spatial probe test (Stage II), both the APP/PS1 POs and WT groups demonstrated more passing times and a greater percentage of time spent at the platform’s original location. In the reverse-place navigation (Stage III) trial from days 380th–383rd, with the platform moving to the opposite side, the APP/PS1 POs and WT groups initially took longer, indicating stronger spatial memory from Stage I and Stage II. Their reverse passing length and escape latency decreased rapidly over time and were significantly lower than those of the Sham APP/PS1 mice at the last day of this stage. On day 384th, in the reverse spatial probe (stage IV) test without the platform, A_40_-POs treated mice still outperformed the Sham APP/PS1 group. After 180 days, the mice were reperformed for place navigation and spatial probes (Stages V and VI). The conditions were consistent with those of stage I and stage II. Six months after the injection of A_40_-POs, the mice could still find the escape platform within a shorter time in the navigation experiment. They stay longer and traverse more times at the correct location in the spatial probe test. The performance of the mice injected with A_40_-POs (6 months p.i.) was close to that of the wild-type mice with same age, and better than that of the Sham APP/PS1 group (**a**). Place navigation trials (Stages I, III and V) were analyzed via two-way ANOVA, whereas spatial probe trials (Stages II, IV, and VI) were compared via one-way ANOVA. Significance levels are denoted as *p < 0.05, **p < 0.01, ***p < 0.001, ****p < 0.0001, with *n* ≥ 11 (for all 12-month-old mice) and *n* ≥ 6 (for all 18-month-old mice). Nest-construction images (**b**), nest scores (**c**) and sucrose preferences (**d**) were recorded for the APP/PS1 Sham, APP/PS1 POs and wild-type groups at two time points post-injection. (groups were compared via one-way ANOVA. Significance levels are denoted as *p < 0.05, **p < 0.01, ***p < 0.001 with *n* = 5, the data are presented as the means ± SEMs.)

Finally, we investigated the effects of A_40_-POs administration on animal cognition via Morris water maze. As indicated in Fig. 5a the stage I, with the number of experimental days increased, the time they took to find the platform gradually decreased, suggesting that animals made progress in learning and remembering the platform’s location.

The APP/PS1 POs (A_40_-POs-treated) group exhibited a significantly shorter escape path length than the sham-operated APP/PS1 group after training (Fig. 5a, Stage I), suggesting improved spatial navigation strategies. Their search efficiency was similar to that of the wild-type mice. The escape latency (time taken to reach the escape platform) of the APP/PS1 POs group was also shorter than that of the sham group. A shorter escape latency indicates better spatial learning and memory ability. Although the relationship between swimming speed and spatial learning and memory abilities is weak, analyzing swimming speed can rule out the impact of an animal’s motor ability or fear during experiments. For all the parts, there was no significant difference in the swimming speeds of the three groups of mice.

When the platform was removed in Stage II, the APP/PS1 POs group crossed the platform more times and spent a significantly greater percentage of time at the platform’s original location than the Sham group did, reflecting stronger memory of the platform’s location. In Stage III, the escape platform was placed on the opposite side of its original location. Initially, longer search times reflect the long-term memory of the original platform’s location. Nevertheless, as the number of training sessions increased, the group with stronger learning abilities would present a greater reduction in path length and escape latency. In the last two days of this stage, animals treated with A_40_-POs and the wild-type group presented shorter escape paths and escape latencies. When the escape platform was removed (stage IV), the APP/PS1 POs group stayed longer than the sham group at the escape location. This location was crossed more often, reflecting the stronger memory abilities. Six months after the mice were treated with A_40_-POs, we performed this water maze experiment to evaluate the persistence of cognitive improvement in the treated mice. Place navigation and spatial probe tests (Stages V and VI) were performed on the mice adhering to the same methods as those implemented in Stages I and II. Between-group comparisons revealed that the cognitive enhancement provided by A_40_-POs treatment persisted in APP/PS1 mice, and A_40_-POs-treated mice demonstrated a level of cognitive similar to that of wild-type mice, which was significantly greater than that of sham APP/PS1 mice.

Enhancing quality of life is a crucial objective in AD treatment and improvement. To assess the life quality of the mice, we conducted nest construction (Fig. 5b, c) and sucrose preference (Fig. 5d) experiments following the Stages IV and VI of the Morris water maze test. Nest-construction behavior is commonly used to evaluate daily activities, fine motor skills, cognition, and emotional state in mice with cognitive impairments. For mice, a high-quality nest provides thermoregulation and predator avoidance, serving as a security indicator that correlates with executive function performance. The treated group exhibited a significantly higher nest-construction score compared to the Sham group. The sucrose preference experiment was conducted to assess the hedonic response of the animals to sweetness by administering a low-concentration sucrose solution. The group treated with A_40_-POs exhibited significantly higher preference scores compared to the sham APP/PS1 group (Fig. 5d).

Overall, the results of the behavioral experiments indicated that animals treated with A_40_-POs presented improved memory and learning capabilities, enhanced cognition, and elevated quality of life.

## Discussion

The profound therapeutic recalcitrance of late-stage AD arises from a self-reinforcing cascade of neuropathological disturbances: persistent accumulation of Aβ, progressive breakdown of the BBB, collapse of physiological clearance routes, and the convergence of multiple neurodegenerative mechanisms that entrench the disease state.^54–56^ In the classical APP/PS1 mouse model, at 12 months of age, perivascular amyloid deposits accumulate in parallel with a marked reduction in LRP1–endothelial co-localization, signaling the decoupling of the neurovascular unit and the erosion of Aβ efflux capacity. This pathological uncoupling is accompanied by a molecular shift from physiological PACSIN2-mediated tubular transcytosis toward Rab5-driven degradative endocytosis, establishing a microenvironment in which the very barrier dysfunction amplifies amyloid burden, it helps to perpetuate.^41,51,52^ The resulting landscape is not merely one of impaired barrier integrity but of disrupted and maladapted transport machinery. In this context, effective therapy must extend beyond directly targeting amyloid aggregates, instead restoring the underlying vascular transport architecture that maintains neurovascular homeostasis.

Current therapeutic approaches have often focused on improving transport efficiency by tuning ligand affinity, particularly in transferrin receptor-targeting strategies, which have demonstrated that mid-affinity interactions can outperform high-affinity binding by avoiding lysosomal routing and promoting transcytosis. While such strategies have opened a new chapter in neuropharmaceutical design, they largely remain transport-centric, concerned with getting molecules into the brain rather than repairing the transport systems themselves. In contrast, the approach presented here seeks to correct the pathological shift in endothelial trafficking, re-establishing a healthy balance between receptor recycling and degradation. Our strategy is founded on fundamental biophysical principles of receptor–ligand binding thermodynamics, supramolecular spatial encoding, and membrane trafficking dynamics. Receptor fate is governed by a finely balanced interplay between binding energy, spatial confinement, and membrane microdomain organization: mid-avidity cargo engages PACSIN2-facilitated tubular carriers that maintain physiological clearance, whereas high-avidity ligands promote Rab5-mediated lysosomal degradation, progressively depleting functional receptors. This dichotomy acts as a biological switch, directing LRP1 either toward productive transcytosis or toward degradation. Crucially, these processes are not passive but allosterically sensitive to the geometry, valency, and spatial arrangement of ligands at the nanoscale. To exploit this mechanism, we developed A_40_-POs, LRP1-targeted polymersomes displaying angiopep-2 ligands in a spatially programmed multivalent configuration. Computational modeling guided the spatial organization of ligands to emulate physiological neurovascular engagement, minimizing conformational strain that would otherwise trigger degradative routing. This precise nanoscale architecture thermodynamically stabilizes LRP1 conformations favorable for PACSIN2 recruitment, restoring productive transcytosis by recreating the membrane curvature and receptor clustering dynamics of healthy endothelium. In contrast, A_200_-POs with overcrowded ligands induce pathological Rab5 activation through microdomain disruption and receptor conformational distortion, underscoring the fine balance between therapeutic reactivation and aberrant trafficking.

Our study underscores the transformative potential of multivalent targeting and BBB modulation in treating AD. The deployment of LRP1-targeting polymersomes has facilitated rapid Aβ clearance and initiated significant changes in the BBB, leading to improved cognitive outcomes. This innovative therapeutic paradigm offers a promising pathway for developing effective clinical interventions, addressing vascular contributions to AD, and ultimately enhancing patient outcomes. The therapeutic implications are profound. By reinstating PACSIN2-mediated trafficking, A_40_-POs not only restore clearance but also shift LRP1 homeostasis toward upregulation, counteracting the receptor loss driven by pathological high-avidity amyloid binding. This shift represents a form of vascular reprogramming, in which the endothelium regains its native capacity to manage proteopathic stress. Experimental evidence confirms that treatment initiates a rapid clearance phase within hours, marked by a substantial reduction in cerebral amyloid load and concurrent elevation of plasma Aβ, consistent with restored vectorial efflux. Pathological imaging reveals depletion of insoluble aggregates across the isocortex, while functional assays demonstrate normalization of transporter stoichiometry and re-coupling of the neurovascular unit. Multiplex immunohistochemistry confirms reactivation of vesicular trafficking from the luminal surface toward the parenchyma, representing a spatial inversion of pathological amyloid dynamics. Most notably, these structural and molecular restorations translate into long-lasting cognitive preservation, with treated animals performing indistinguishably from wild-type controls in complex spatial learning tasks over extended observation periods. The conceptual advance here lies in moving beyond the paradigm of “overcoming the barrier” toward “repairing the barrier.” A_40_-POs demonstrate that nanoscale spatial programming can restore the BBB’s intrinsic clearance machinery by directly modulating the conformational and trafficking dynamics of its transport receptors. This represents a transition from nanocarriers as passive shuttles to active supramolecular regulators of endothelial biology. The therapeutic trilogy achieved—amyloid clearance, barrier restoration, and sustained cognitive recovery—establishes a blueprint for precision neurovascular medicine.

While current studies have substantiated the validity of this novel theoretical framework, the application of cell-specific knockout models in mechanistic studies holds significant potential to enhance our understanding of the functional role of the PACSIN2 pathway within the pathophysiological context of AD. Looking ahead, the translational journey will require accounting for interspecies differences in receptor glycosylation and membrane composition, as well as vascular pathologies such as cerebral amyloid angiopathy and pericyte loss, which are incompletely recapitulated in murine models. Strategies integrating spatiotemporal mapping of clearance dynamics with human-specific in vitro models, computational simulations under physiological shear stress, and tailored ligand designs informed by patient-specific LRP1 polymorphisms will be essential. The potential extends beyond AD to Parkinson’s, amyotrophic lateral sclerosis, and other disorders where vascular transport failure accelerates neurodegeneration.

In essence, this work illustrates that the BBB is not merely an obstacle to be bypassed but a dynamic and reparable interface whose dysfunction can be therapeutically reversed. By embedding the principles of receptor thermodynamics and supramolecular spatial encoding into material design, we have demonstrated that pathological trafficking can be reprogrammed toward physiological transport, converting the barrier itself into a therapeutic target. The A_40_-POs paradigm demonstrates that rational materials design integrated with supramolecular engineering can reprogram pathological pathways into targeted therapeutic interventions, establishing precise regulation over endogenous transport systems. This bidirectional engineering framework not only recapitulates natural regulatory paradigms but achieves functional expansion through predictable assembly. This reframing not only changes how we approach drug delivery in neurodegeneration but also heralds a new era in which nanomaterials act as intelligent modulators of cellular behavior, capable of decoding disease-specific transport disruptions and orchestrating their repair at the molecular scale.

## Materials and methods

### A_40_-PO polymersome preparation and characterization

P[(OEG)_10_MA]_20_-PDPA_120_ and angiopep2-P[(OEG)_10_MA]_20_-PDPA_120_ copolymers were synthesized via atom transfer radical polymerization (ATRP) as previously reported.^33^ A_40_-POs were formulated adhering to the film-rehydration method, whereby P[(OEG)_10_MA]_20_-PDPA_120_ and a 1.88% molar ratio of angiopep2-P[(OEG)_10_MA]_20_-PDPA_120_ were dissolved in a mixture of methanol and chloroform (v/v, 3:1). The mixture was left to evaporate, allowing the organic solvent to completely dissipate and form a homogeneous polymer film at the bottom of the vial. PBS solution was added to rehydrate the polymer film and sonicated for 30 min. The solution was stirred continuously for 7 days at 4 °C via a magnetic stirrer at 1000 rpm. The morphology of the A_40_-POs was characterized via transmission electron microscopy (JEM-2100Plus, Japan). The diameter distribution was assessed via dynamic light scattering (Malvern Zetasizer Pro, UK).

### Animals

All the animal studies were conducted in accordance with the guidelines set forth by the West China Hospital Animal Care Committee (IACUC-approved project number: 20211475A). Considerable efforts were made to minimize the number of animals utilized in these studies and to alleviate any pain or discomfort experienced by the animals. In all the experiments, the animals were housed in a room where the temperature was regulated, with consistent alternating cycles of light and darkness.

### Immunohistochemistry (IHC)

Paraffin-embedded sections (5 μm) of mouse brain tissue were deparaffinized with xylene and subjected to gradient alcohol hydration (100%, 80%, 50%, 30%). Endogenous peroxidase activity was quenched via the addition of 3% H_2_O_2_ (room temperature, 10 min) following antigen retrieval (Biosharp, 22315828). The tissues were blocked with 1% BSA (Aladdin, A104912) at room temperature for 30 min. Aβ-targeting primary antibodies were incubated overnight at 4 °C (Servicebio, GB13414-1, 1:200), followed by washing with PBS. The sections were then incubated with HRP-conjugated secondary antibodies (ABclonal, AS014, 1:150) at ambient temperature for 30 min and subsequently washed with PBS. DAB substrate (Elabscience, E-IR-R101, 1:20) was applied for 5 min, followed by hematoxylin solution (Servicebio, G1004) for 1 min and hematoxylin differentiation solution (Servicebio, G1039) for 10−15 s. The sections were immediately rinsed in running tap water for 20 s, treated with hematoxylin bluing buffer (Servicebio, G1040) for 1 min, and washed with PBS. Finally, the slides were mounted with quick-drying neutral resin (ZSZSGBBIO, ZLI-9516).

### Aβ extraction

Following euthanasia via an overdose of isoflurane anesthesia, cardiac perfusion was conducted with cold PBS. The entire brain tissue was subsequently harvested and weighed. The brain tissues were subjected to comprehensive homogenization (Servicebio, KZ-5F-3D) employing a TBS solution enriched with phosphatase inhibitor (Servicebio, CR2302054) and protease inhibitor (Servicebio, CR2306008) at 70 Hz and −20 °C. The supernatant was segregated after the brain tissue homogenate was centrifuged for 1 h at 100,000 × *g* and 4 °C via an ultrahigh-speed centrifuge (Beckman, Optima MAX-XP). The sediment were subsequently resuspended in 70% formic acid (Aopusheng (Tianjin) Chemical, 20210610) and centrifuged at 100,000 × *g* and 4 °C for 1 h, after which the resulting supernatant was collected.

### Vessel extraction

Immediately following euthanasia via isoflurane overdose anesthesia, cardiac perfusion was conducted with cold PBS, then brain tissues were excised and weighed. The tissues were processed via vascular parenchyma isolation buffer comprising 10 mM HEPES (Servicebio, CR2207064), 141 mM NaCl (Aladdin, 111549), 4 mM KCl (Aladdin, P112134), 2.8 mM CaCl_2_ (Aladdin, C290953), 1 mM MgSO_4_ (Aladdin, M433513), 1 mM NaH_2_PO_4_ (Aladdin, S433623), and 10 mM glucose (Servicebio, CR2112094). To 500 μL of brain tissue homogenate, 1 mL of 26% (w/w) dextran (Next Sage, 61212ES60) was added. Following thorough mixing and a resting period of 15 min at 4 °C, the mixture was centrifuged (15,800 × *g*, 15 min, 4 °C). The upper layer subsequently represented the parenchymal fraction, and the lower layer corresponded to the vascular fraction. The vascular fraction was washed with PBS 2 × 5 min for further detection.

### Enzyme-linked immunosorbent assay (ELISA)

The parenchyma and blood vessels underwent comprehensive homogenization using a tissue lysis mixture (Yase, 016c1050) enriched with a phosphatase inhibitor (Servicebio, CR2302054) and a protease inhibitor (Servicebio, CR2306008) at 70 Hz and −20 °C. The tissues were allowed to fully lyse at 4 °C for 15 min. The supernatant was collected via centrifugation at 14,000 × *g* and 4 °C for 10 min. The protein concentrations of various proteins, including LRP1 (NOVUS, NBP3-00449), PACSIN2 (Anruike, YX-160103M), Rab5 (abbexa, abx154598), and Aβ (Invitrogen, KMB3441), were determined via ELISA kits following the manufacturer’s protocols. For Aβ in the blood, the test was performed directly according to the ELISA instructions. Aβ extracted by formic acid needs to be neutralized with Tris base before testing. Protein concentrations were calculated on the basis of the curve equation (four-parameter fit).

### Confocal imaging

Confocal images were captured via a Leica Stellaris 5 confocal microscope equipped with Diode 405, Argon, DPSS 561, and HeNe633 lasers. Imaging was conducted at a resolution of 2048 × 2048 pixels and a scanning speed of ×100. Colocalization analysis to derive PCC (r) was performed via the colocalization plug-in for ImageJ.

### STED imaging

STED nanoscopy experiments were performed under a Leica DMi8 confocal microscope equipped with a Leica TCS SP8 STED-ONE uni. The dyes (TSA570, TSA620 and TSA750) were excited under an STED laser. The emission signals were collected via HyD reflected light detectors. The depletion beam was applied at wavelengths of 592 nm, 660 nm, and 775 nm (50% power), with a resolution of 2048 × 2048 pixels and a scanning speed of ×100.

### Tyramide signal amplification (TSA) stain

Mouse brain tissue sections were deparaffinized with xylene and hydrated through gradient alcohol dehydration. Following antigen retrieval, 3% H_2_O_2_ was utilized to inactivate endogenous peroxidase (Biosarp, 22315828) for 10 min. Permeabilization of the sections at room temperature for 10 min was achieved with 1% Triton X-100 (Biofroxx, 1139ML100). The sections were rinsed with PBS and incubated with the primary antibody for 1 h at room temperature. After washing with PBS, the samples were incubated with the HRP-conjugated secondary antibody for 10 min. Subsequent to a PBS rinse, the TSA fluorescent solution (Absin, abs50031) was applied and incubated at room temperature for 10 min. Antigen retrieval was subsequently conducted. The aforementioned steps were repeated with distinct primary antibodies, including antibodies against LRP1 (ABclonal, A1439, 1:100), Aβ (Servicebio, GB13414-1, 1:200), CD31 (Cell Signaling, 77699, 1:300), Rab5 (Thermo Fisher, PA5-88260, 1:200) and CD146 (Abcam, ab75769, 1:300). Various wavelengths of TSA, including the TSA520, TSA570, TSA620, and TSA700 dyes, until multimeric fluorescence staining was achieved. The nuclei were stained with DAPI (Solarbio, C0065) for 5 min and then sealed with antiquenching sealing agent (Solarbio, S2100).

### Positron emission tomography-computed tomography (PET-CT) imaging

APP/PS1 POs (12-month-old) mice were injected intravenously with the commercial Aβ radiocontrast agent [^18^F] AV-45 (2.8–3.2 MBq). Then, intracranial images of the mice were acquired via a micro-PET-CT imager (Inviscan, France) according to operation specifications. After a recovery period, saline (200 μL) was intravenously injected into wild-type mice (3- and 12-month-old) and sham APP/PS1 mice (12 months old). The APP/PS1 POs were intravenously injected with 200 μL of A_40_-POs (10 g/L). After 12 h, all the mice were intravenously injected with [^18^F] AV-45 (2.8–3.2 MBq). Intracranial images of the mice were acquired via the same imager.

### Hematoxylin‒eosin (H&E) staining

Mice tissue sections (5 μm) were dewaxed and rehydrated. The sections were stained with hematoxylin solution for 5 min, immediately rinsed in running tap water for 10–15 s, followed by incubation with hematoxylin differentiation solution (Servicebio, G1039) for 10–15 s. Subsequently, the sections were treated with hematoxylin bluing solution (Servicebio, G1040) for 30 s and washed in running tap water. The sections were then stained with eosin staining solution for 30 s, followed by washing with ethanol. The slides were dehydrated through a gradient of ethanol (100%, 80%, 50%, 30%) and xylene. Seal the sections with quick-drying neutral resin (ZSZSGBBIO, ZLI-9516).

### Tissue clearing and staining

The paraformaldehyde-fixed mouse brain were treated with 1/2 CUBIC-L solution (80 mL Milli-Q, 5 g Triton X-100, and 5 g N-butyldiethanolamine (Aladdin, 102-79-4)) at 37 °C for 6 h. Then, the solution was replaced with a CUBIC-L solution and incubated at 37 °C for 15 days, with the new CUBIC-L solution being replaced every 2 days. The brain tissues were subsequently washed three times with staining buffer (1.5 M NaCl). The hyalinized brains were transferred to staining buffer containing a vasculature probe (Vector, DL-1178-1, 1:100) and an Aβ probe (Abcam, ab216983, 100 nM) for fluorescence staining (RT, 3 days). The refractive index was adjusted via a CUBIC-M solution (25 g Milli-Q, 45 g antipyrine (Aladdin, 60-80-0), 30 g N-methylnicotinamide (Aladdin, 114-33-0), and 125 μl of N-butyldiethanolamine)). Images were analyzed via Amira and iMaris software.

### High-performance liquid chromatography (HPLC)

Donepezil HCl (Aladdin, D129948) was dissolved and added to a polymer film preparing donepezil HCl@A_40_-POs, followed by dialysis using a 3 kDa dialysis bag for 7 days. The concentration of donepezil HCl in the dialysis fluid was measured to calculate the encapsulation efficiency. Sodium 1-decanesulfonate (Aladdin, S100284) was dissolved in pure water (15.7416 mM/L) and filtered. Chromatographic grade acetonitrile solution and perchloric acid were added, followed by ultrasonication for 10 min. Standard solutions of donepezil HCl at concentrations of 250, 125, 62.5, 31.25, 15.625, and 7.8125 μg/mL were prepared. The content of donepezil HCl was detected via an Agilent-1260 chromatograph at a flow rate of 1 mL/min, a column temperature of 35 °C, a volume of 20 μl, and a detection signal at 271 nm.

### Morris water maze (MWM) experiment

In each group, the mice were administered a caudal vein injection daily (A_40_-POs, donepezil@A_40_-POs, donepezil, or saline), which was continued for three days. The mice were subsequently housed in a standard rearing environment for seven days to acclimatize and recover. For the analysis, the pool was segmented into four quadrants. From days 11 to 14 (days 375–378 of lifespan), a platform was positioned in quadrant II, and the animals were introduced into the thermostatic pool from each quadrant daily, with time taken by the mice to locate the platform recorded as escape latency. If the mice failed to reach the platform within 120 s, they were guided to it and remained there for 30 s. A spatial probe (Stage II) was performed on day 15 (day 379 of lifespan). Once the platform was removed, the mice were placed in the water from the IV quadrant. The results of the spatial probe were expressed as either the percentage of time the mice remained at the original escape platform location or the number of times they passed. Reverse place navigation trials were conducted from days 16 to 19 (day 380–383 of lifespan), with the platform positioned in the IV quadrant which opposite the original platform location. The animals repeated the regimen from days 11 to 14, and the reverse escape latency was documented. On day 20 (day 384 of lifespan), the platform was removed, and the mice were introduced into the water from quadrant II. The results of the reverse spatial probe are expressed as either the percentage of time that the animal spent on the original platform position or the number of times it crossed the location. In addition, 6 months later, the mice were subjected to place navigation (stage V) and spatial probe (stage VI) experiments again with the same methods and conditions. Animal performance was recorded by the same tracking system (EthoVision XT, Noldus Information Technology) for each stage.

### Sucrose preference

At the end of Stage IV and Stage VI, the sucrose preference of the sham, APP/PS1 and APP/PS1 POs was tested. Each mouse was housed individually and allowed to acclimatize to the cage, which contained two bottles of standard pure water, for 2 days. One of the bottles was subsequently filled with 2% sucrose solution. The amount of water consumed by the mice was recorded, and the water in each bottle was refreshed daily. Sucrose preference = Sucrose water consumption/(Sucrose water consumption + Standard purified water consumption) × 100%

### Nest construction

Three days after the sucrose preference experiment, each mouse was housed individually and then adapted to a single-cage environment for 7 days. Subsequently, 10 pieces of paper (5 × 5 cm^2^) were added to each cage and evenly placed. After 4 days, the nesting test results were scored in accordance with the improved 4-point scoring system.^57^ 1 point, no visible tear, no recognizable nest site; 2 points, no visible tear, nest site recognizable; 3 points, partial tear, recognizable nest site; 4 points, sharpest tear, recognizable nest.

### Digital western blot

Western blot analysis was performed via the Jess automated digital system (ProteinSimple, Santa Clara, CA, USA) with 12–230 kDa separation modules. Total protein concentrations were determined via a bicinchoninic acid (BCA) assay (Biosharp, BL520A). The samples were diluted in 1× sample buffer and 5× master mix (ProteinSimple) to a final loading concentration of 1 μg/μL, denatured at 95 °C for 5 min. They were then loaded into wells at a volume of 3 μL per well. Denatured samples, blocking buffer, primary antibodies, HRP-conjugated secondary antibodies (Jess modular antibodies, ProteinSimple), wash buffer, and chemiluminescent substrate (1:1 luminol-peroxidase mixture) were sequentially loaded into the designated detection plate wells. The primary antibodies used included rabbit anti-CD31 (1:50; Cell Signaling Technology, #77699), anti-CD146 (1:50; Abmart, T55209), anti-NeuN (1:50; Abcam, ab177487), and anti-β-actin (1:300; Servicebio) antibodies. Automated capillary electrophoresis, immunoblotting, and signal acquisition were performed via the Jess system according to operation specifications. The data were analyzed with Compass for Simple Western software (v6.2, ProteinSimple).

### Proximity ligation assay (PLA)

Protein‒protein interaction analysis was performed using the Duolink® PLA Kit (Sigma‒Aldrich; DUO96000 and DUO96040) strictly following the manufacturer’s operational protocol. The brain slices of the mice were pre-stained with TSA dye for pericytes (CD146, Abcam, ab75769), endothelial cells (CD31, Cell Signaling, 77699), and astrocytes (GFAP, Invitrogen, PA5-16291). After sodium azide neutralization pre-treatment, the antibodies, LRP1 (Abcam, ab92544), PACSIN2 (Invitrogen, PA5--99032), and Aβ (ABclonal, A24422), were conjugated to species-specific PLA probes (DUO96040). Subsequent procedures inclouding incubation, hybridization, ligation, amplification, and signal development—were conducted according to the kit’s standard protocol. Nuclei were counterstained with DAPI, and images were acquired via confocal microscopy.

## Supplementary information

Supporting data

## Data Availability

All data supporting the findings of this study are included in the main text and supplementary materials. Data can be accessed from the corresponding author upon reasonable request, subject to compliance with consent agreements and applicable data usage restrictions.
