# Supplementary material for: Rapid amyloid-β clearance and cognitive recovery through multivalent modulation of blood–brain barrier transport
Source: Signal Transduct Target Ther. 2025 Oct 7;10:331. doi: 10.1038/s41392-025-02426-1 (PMC12500928; doi:10.1038/s41392-025-02426-1)
Supplement: Supplementary file 1 — Supporting data [file 41392_2025_2426_MOESM1_ESM.docx]

Supplementary Materials for

Rapid amyloid-β clearance and cognitive recovery through multivalent modulation of blood–brain barrier transport

Junyang Chen, **^1,2,3,4,7#^** Pan Xiang, **^1,2,#^** Aroa Duro-Castano, **^4,10^** Huawei Cai, **^1^** Bin Guo, **^5^** Xiqin Liu, **^1^** Yifan Yu, **^1^** Su Lui, **^1^** Kui Luo, **^1^** Bowen Ke, **^1^** Lorena Ruiz Perez, **^1,3,4,6^** Qiyong Gong, **^1,5,8*^** Xiaohe Tian**^1,2,5,7 *^**, Giuseppe Battaglia**^1,3,4,9*^**

Correspondence to: Qiyong Gong (qiyonggong@hmrrc.org.cn), Xiaohe Tian (xiaohe.t@wchscu.cn), and Giuseppe Battaglia ([gbattaglia@ibecbarcelona.eu](mailto:gbattaglia@ibecbarcelona.eu))

**This file includes:**

Supplementary Fig. S1 to S14 with legends

**
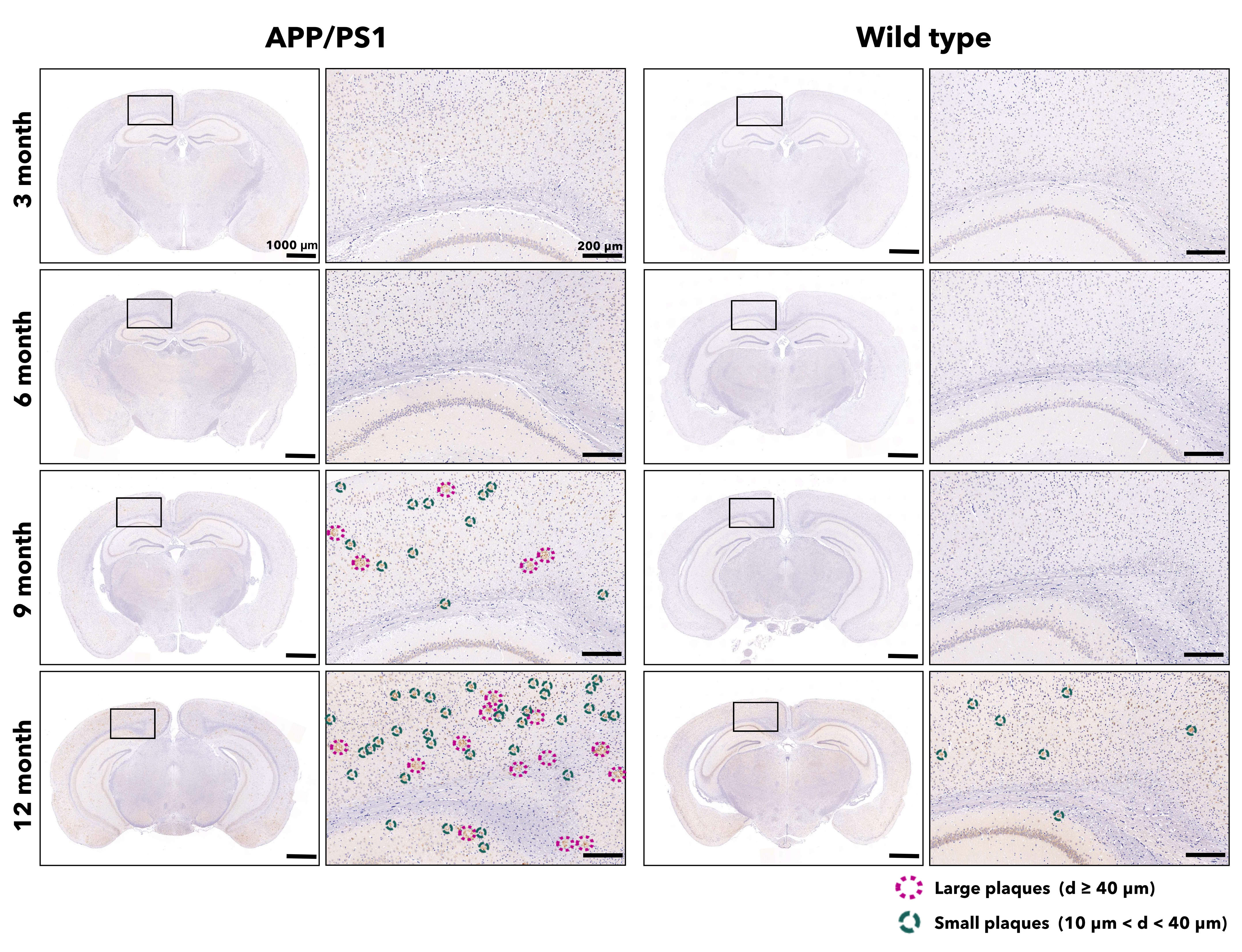
****Figure. S1.** **IHC images of Aβ in the brains sections of age-matched mice.**

IHC images of Aβ in coronal brain sections from mice at 3, 6, 9, and 12 months old. Each section is derived from a different mouse brain. As age increases, area percentage of the brain occupied by Aβ increases in both APP/PS1 and wild type mice (n = 3).

**
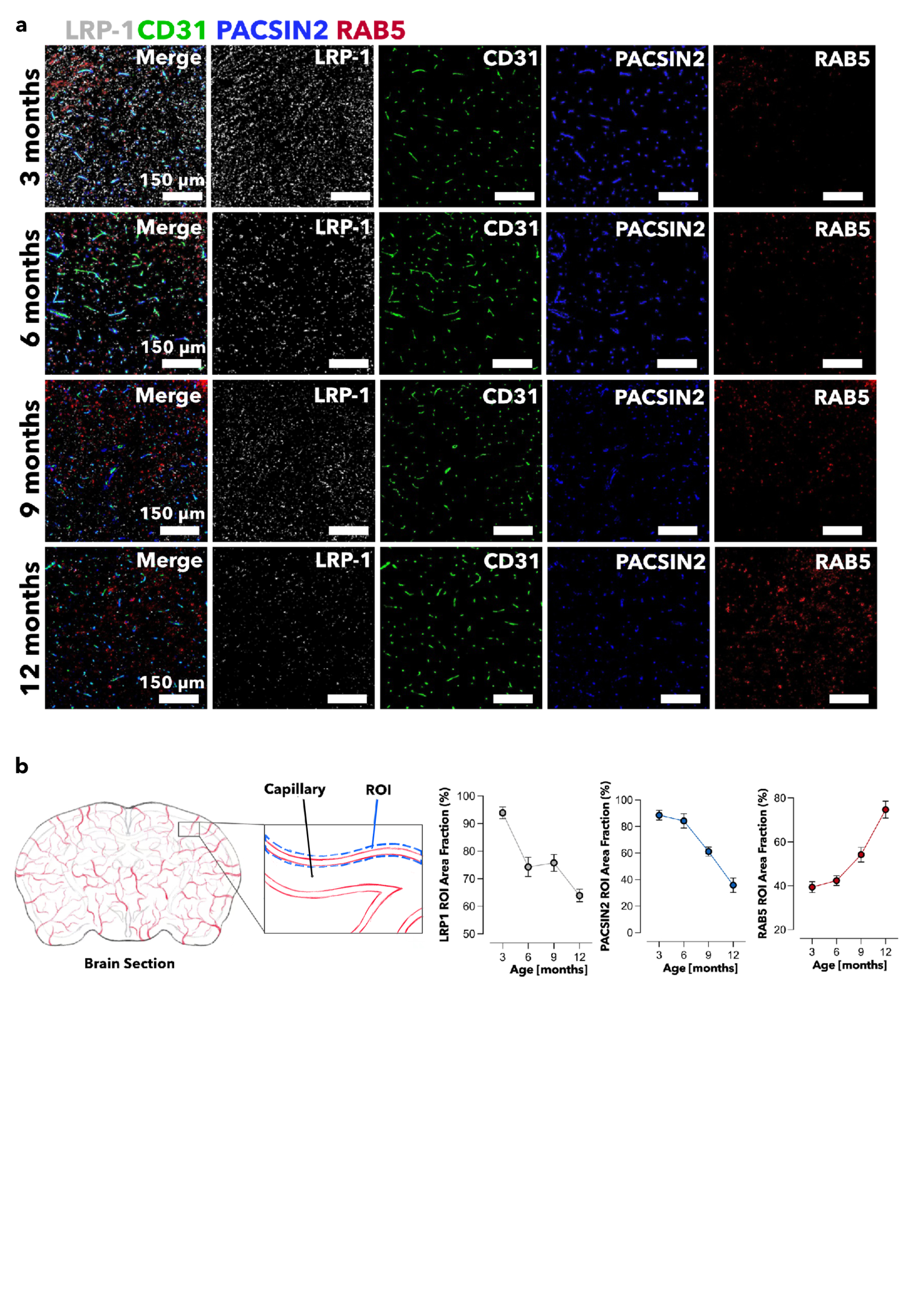
Figure. S2. Fluorescence micrographs colocalization analyzis of age-matched brain sections.**

Fluorescence images of brain sections from 3, 6, 9, and 12 months old wild-type mice, LRP1 (grey) CD31 (green) PACSIN2 (blue) Rab5 (red) (**a**). Capillary regions were counted as ROIs, and the percentage area of LRP1, PACSIN2, and RAB5 in the ROIs was counted. A total of 10 capillaries from two independent trials were selected for counting. LRP1, PACSIN2, and Rab5 showed the same trend as in the ELISA assay (**b**).

**
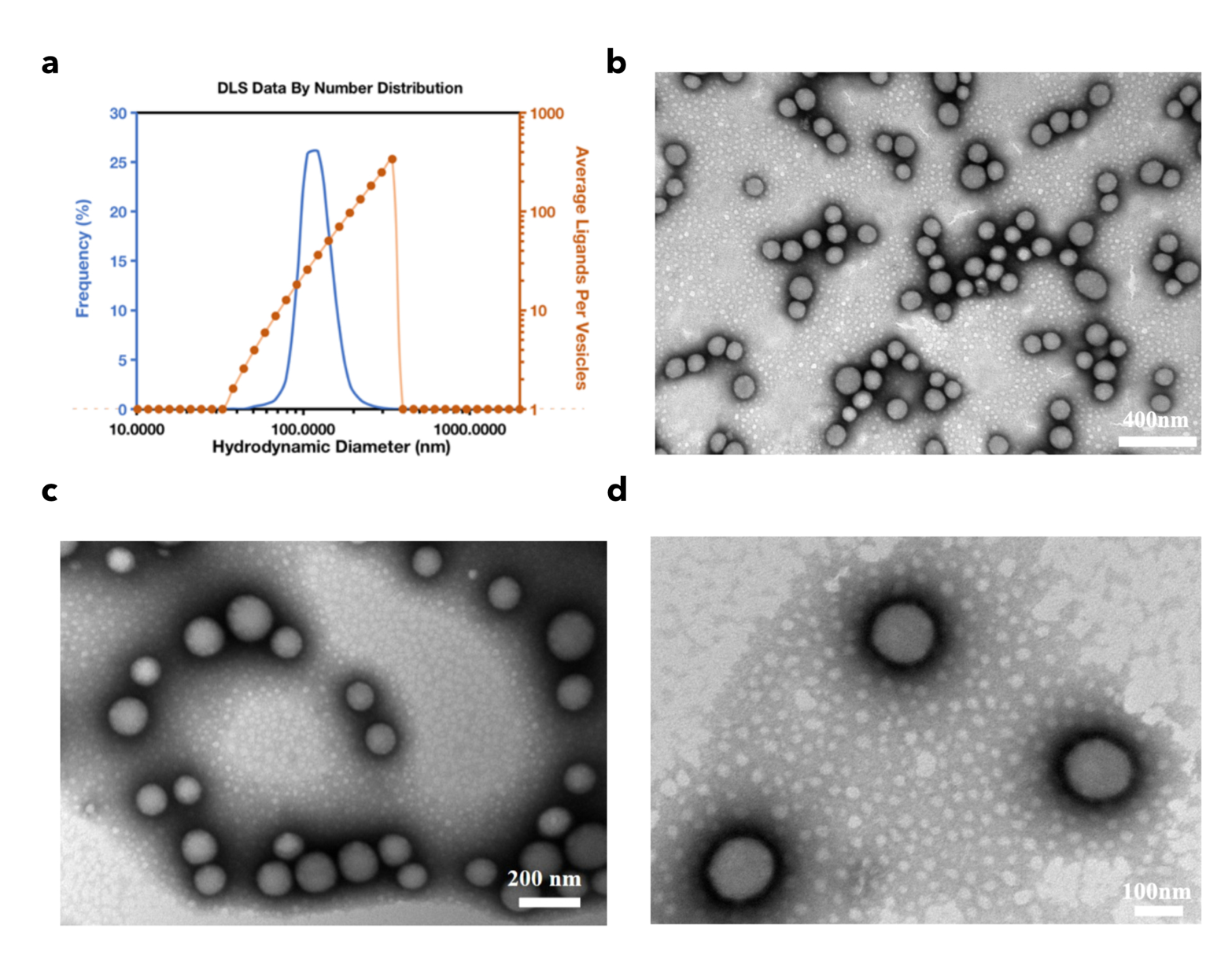
Figure. S3. Characterization of A_40_-POs in terms of size and morphology.**

Size distribution and diameter matched ligands number of A_40_-POs (**a**). TEM images of A_40_-POs magnified × 60,000 (**b**), × 100,000 (**c**) and × 140,000 (**d**).

**
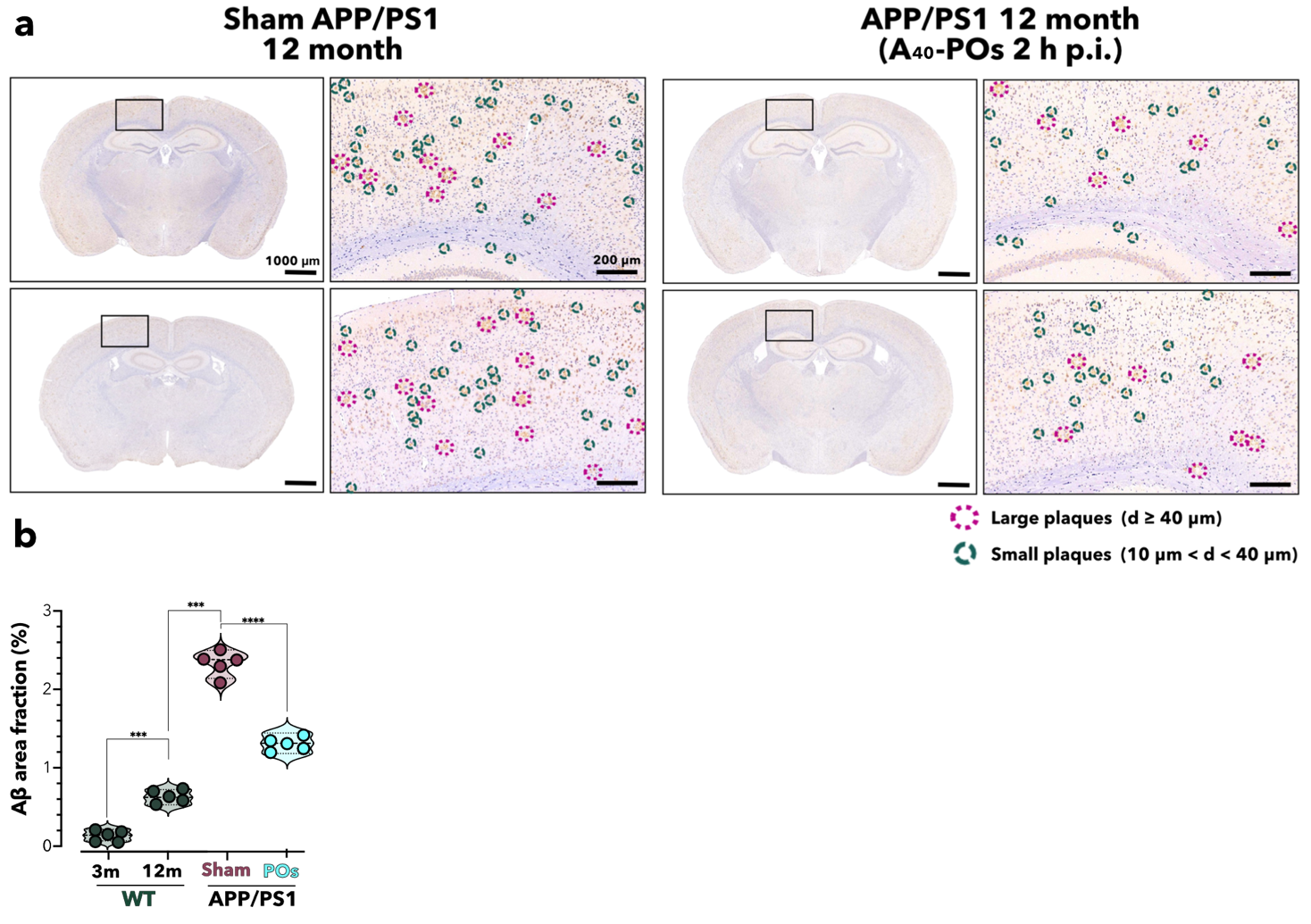

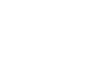
****Figure. S4. IHC images of Aβ in the brain sections of 12-month-old Sham APP/PS1 and A_40_-POs treated mice.**

IHC images of Aβ in coronal brain sections from 12-month-old mice. Each section is derived from a different mouse brain. The percentage of the brain area occupied by Aβ plaques reduced after 2 h post A_40_-POs injection. Meanwhile, both the number of large and small plaques decreased (**a**). The Aβ plaque-positive area fraction in the whole coronal sections of wild-type mice at 3- and 12-months-old (representative images in Fig. S1), APP/PS1 mice at 12 months of age, sham-treated controls and A_40_-POs 2 h post-injection mice (**b**).
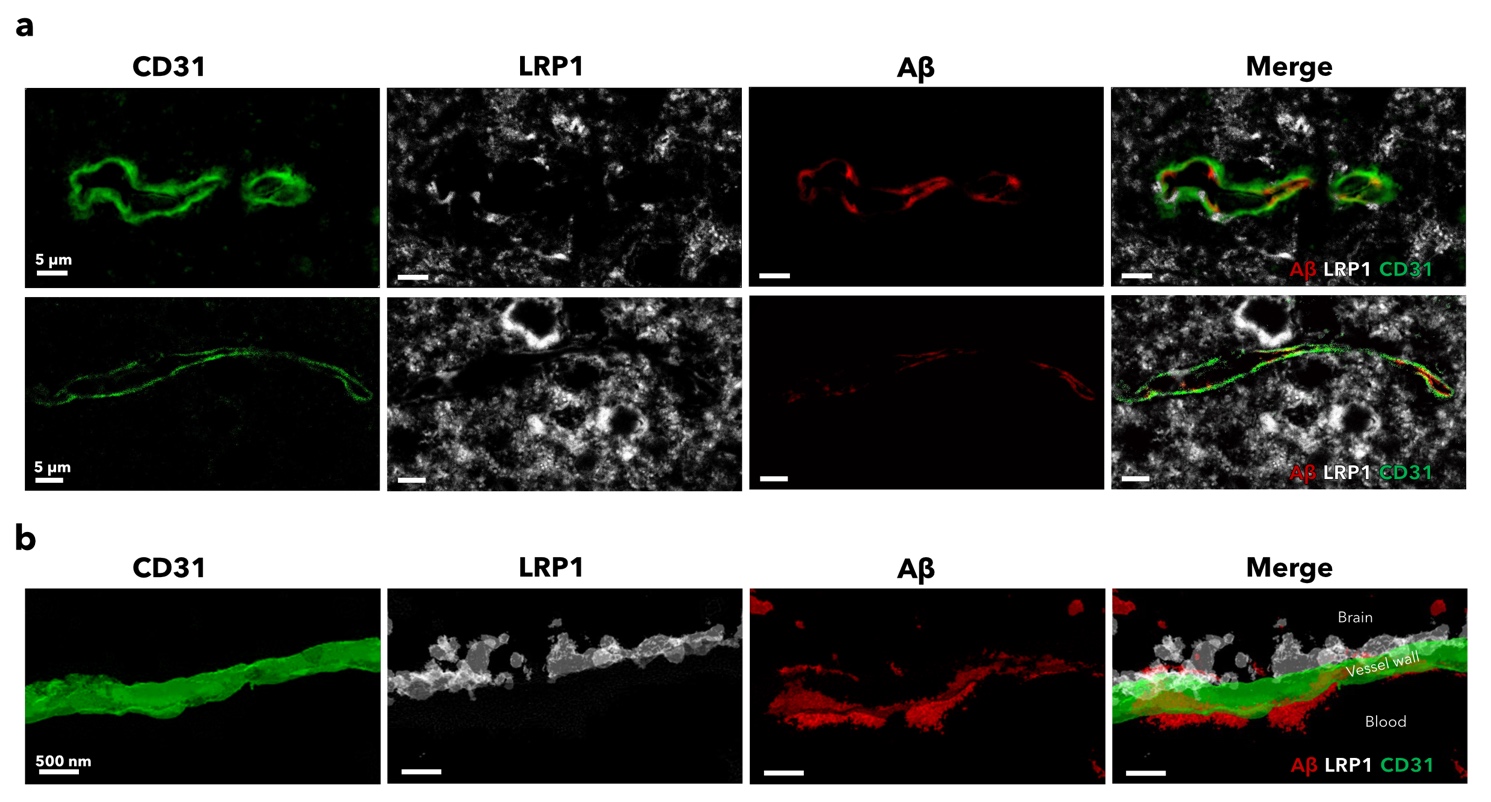
**Figure. S5. Confocal and STED microscopy images depicting the vascular clearance of Aβ.**

Confocal microscopy (**a**) and STED microscopy (**b**) of LRP1 (white) on the vessel wall (green), indicative of active transcytosis. Post-treatment, Aβ deposits around the BBB (red) are cleared with notable Aβ signal presence within the vascular lumen. (The channel merged STED image is also shown in Fig. 4e)

**
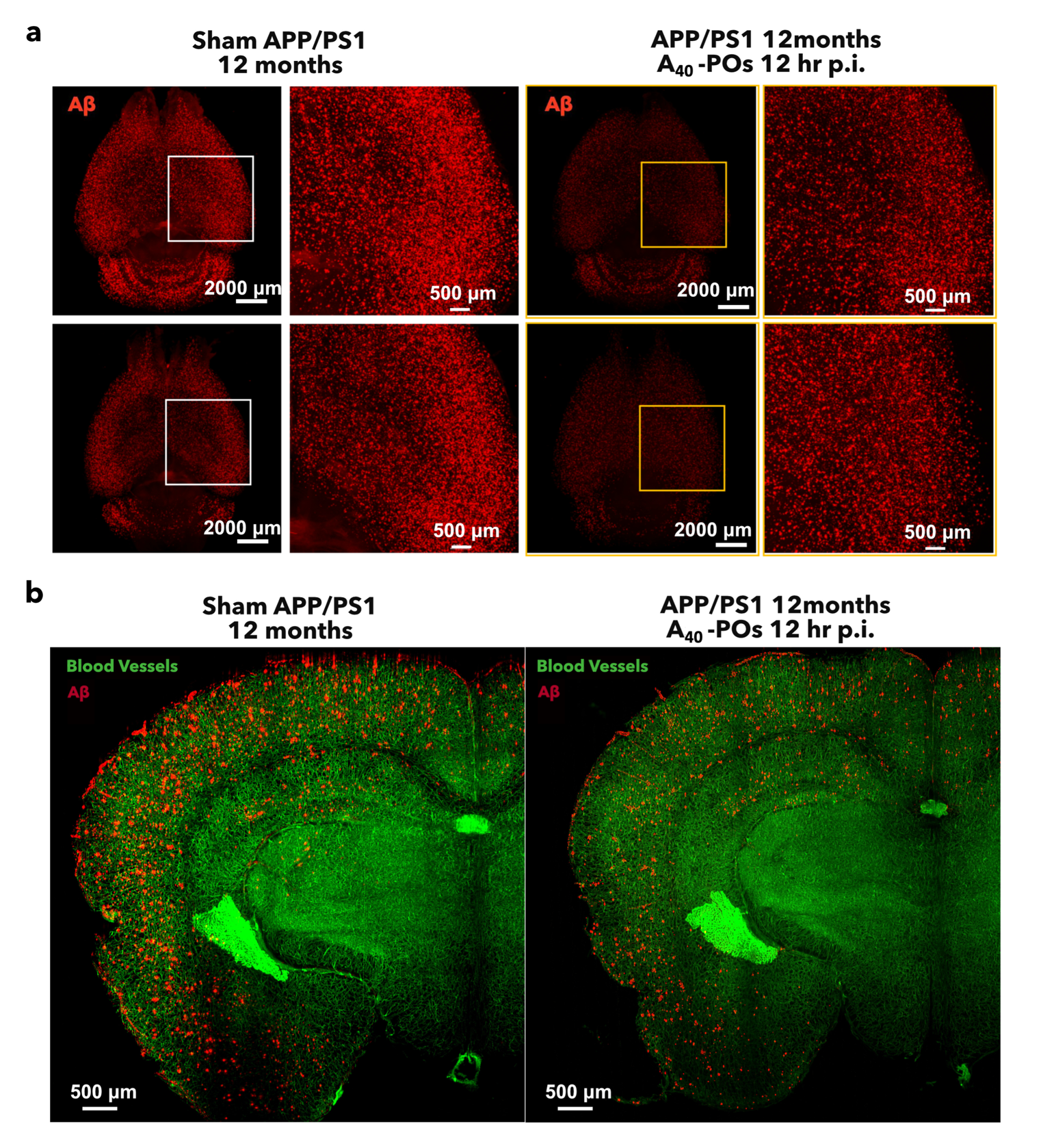
Figure. S6. Brain clearing images of 12-month-old Sham APP/PS1 and A_40_-POs treated mice.**

3D brain clearing images of Aβ (red) in the mice brain (**a**). Coronal view of mouse brain with a thickness of 300 μm (BBB in green, Aβ in red) (**b**). The Aβ signal is attenuated in the brains treated with A_40_-POs.

**
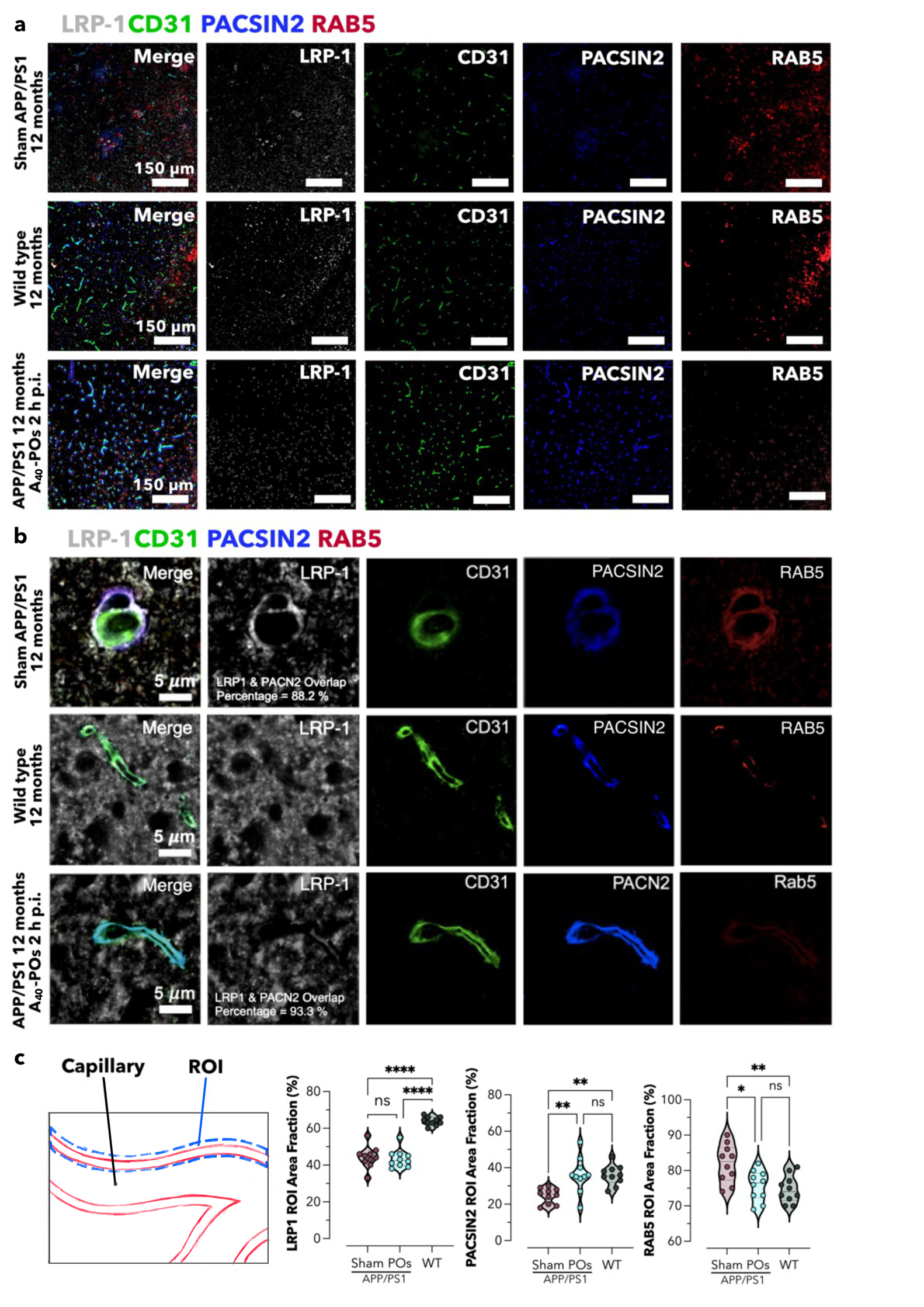
**

**Figure. S7. Fluorescente micrographs co-loclization analysis of treated brain.**

Fluorescence images of brain sections from 12-month-old wild-type, AD, and treated AD mice, LRP1 (grey) CD31 (green) PACSIN2 (blue) Rab5 (red) (**a**). Confocal microscope image of brain sections from 12-month-old wild-type AD and treated AD mice (**b**). Capillary regions were counted as ROIs, and the percentage area of LRP1, PACSIN2, and Rab5 in the ROIs was counted. A total of 10 capillaries from two independent trials were selected for counting. LRP1, PACSIN2, and Rab5 followed the same trend as in the ELISA assay (**c**)


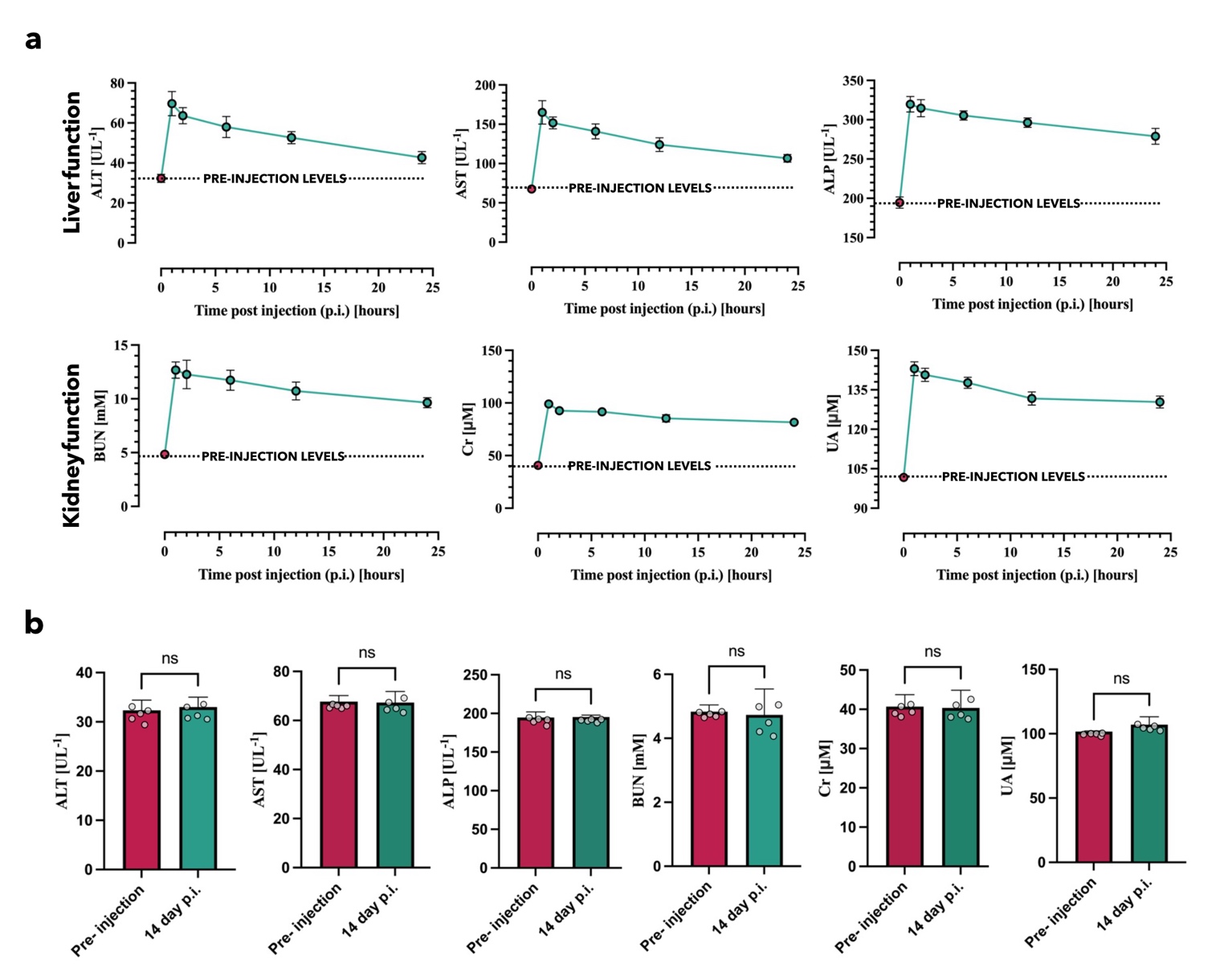
**Figure. S8. Blood biochemical tests to evaluate the biosafety of A_40_-POs.**

Liver (ALT, AST, and ALP) and Kidney (BUN, Cr, and UA) function test of A_40_-POs treated mice at post-injection time points of 1 hour, 2 hour, 6 hour, 12 hour, and 24 hour (**a**) and 14 days (**b**).


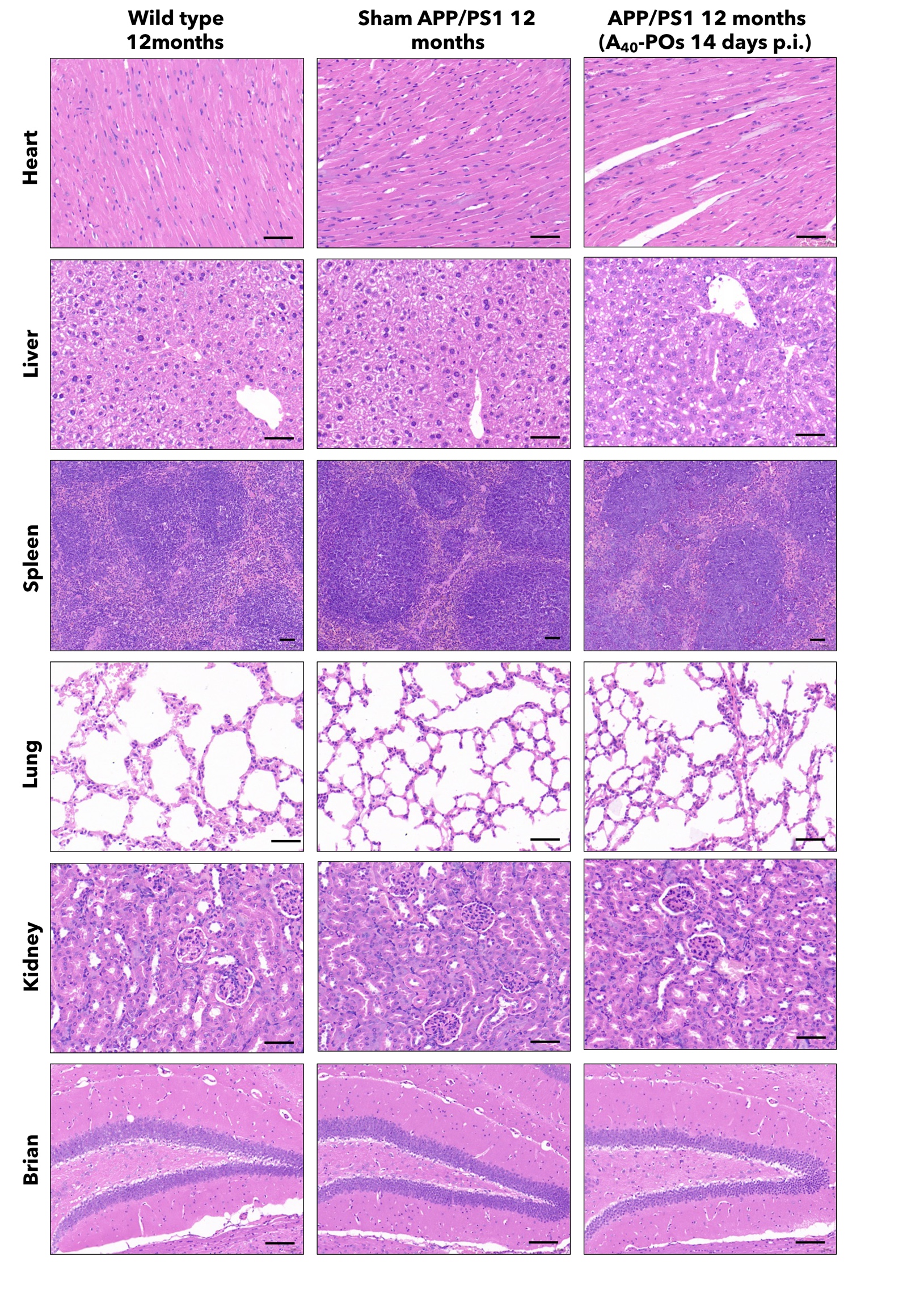


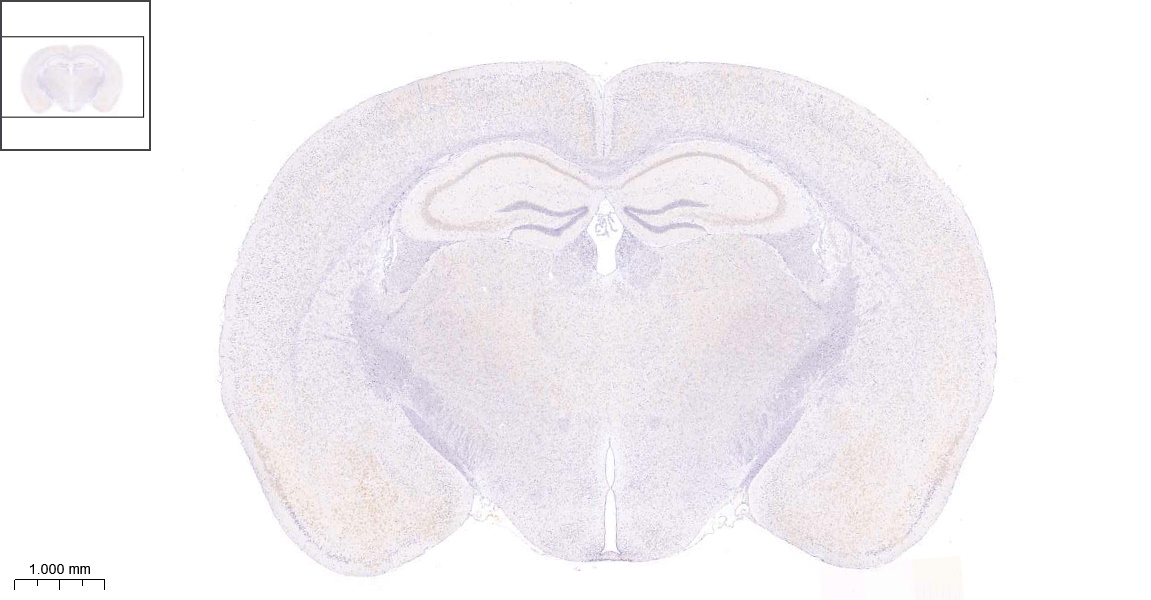


**AD 3 mon**


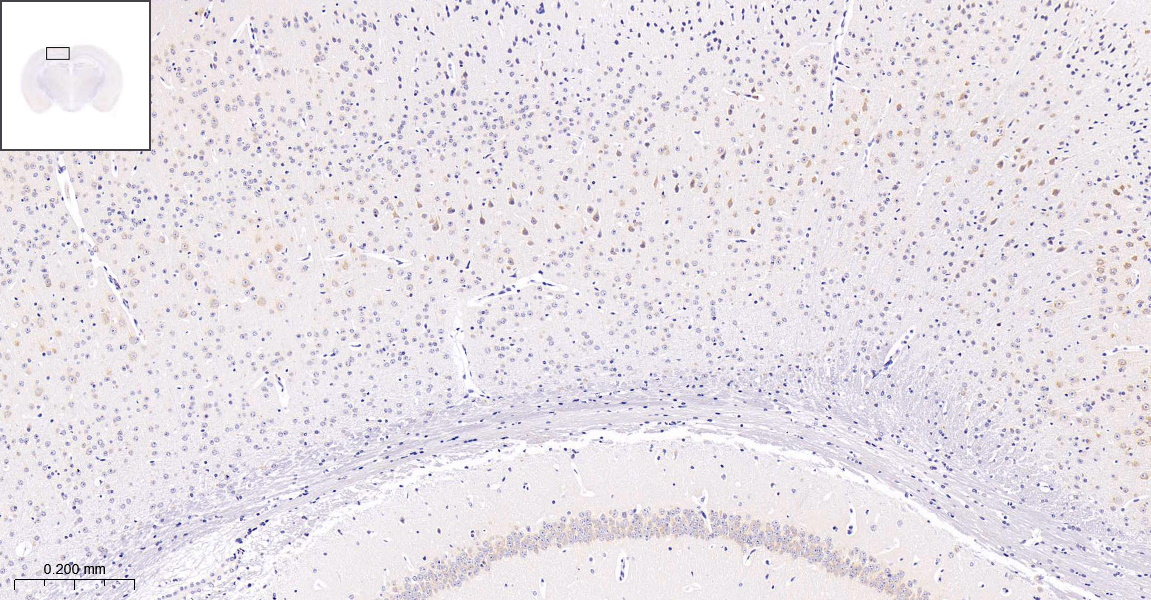

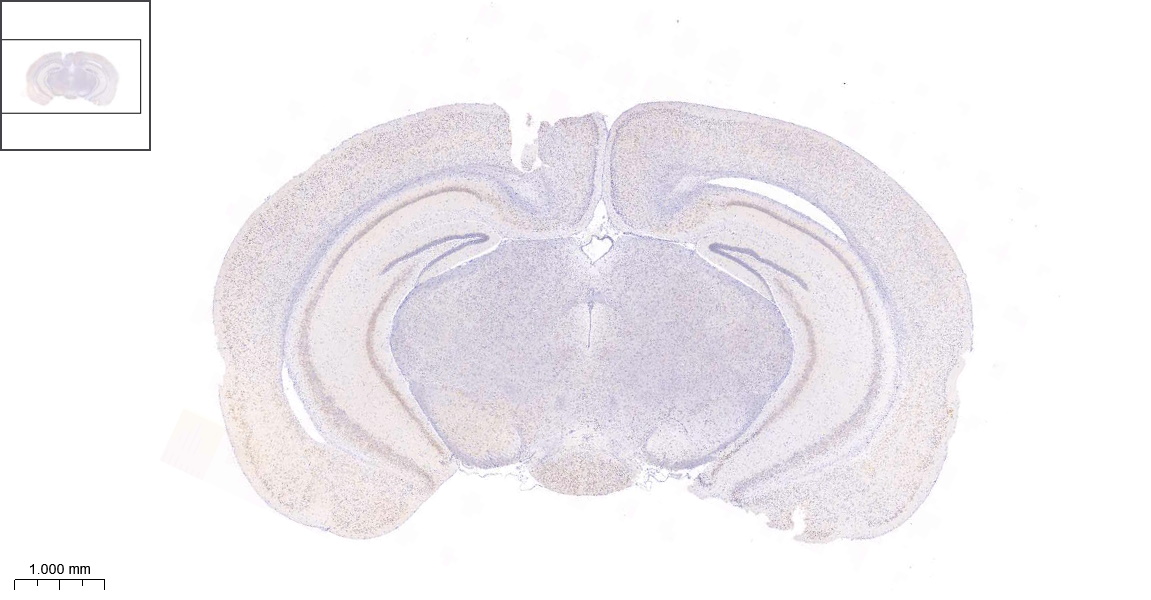


**AD 3 mon**


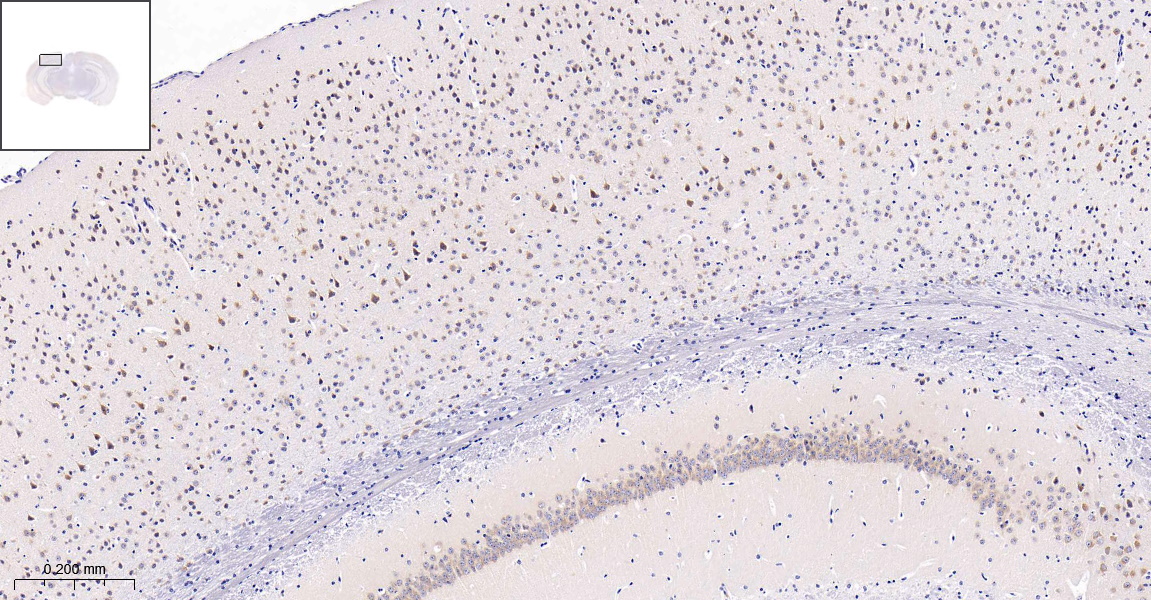

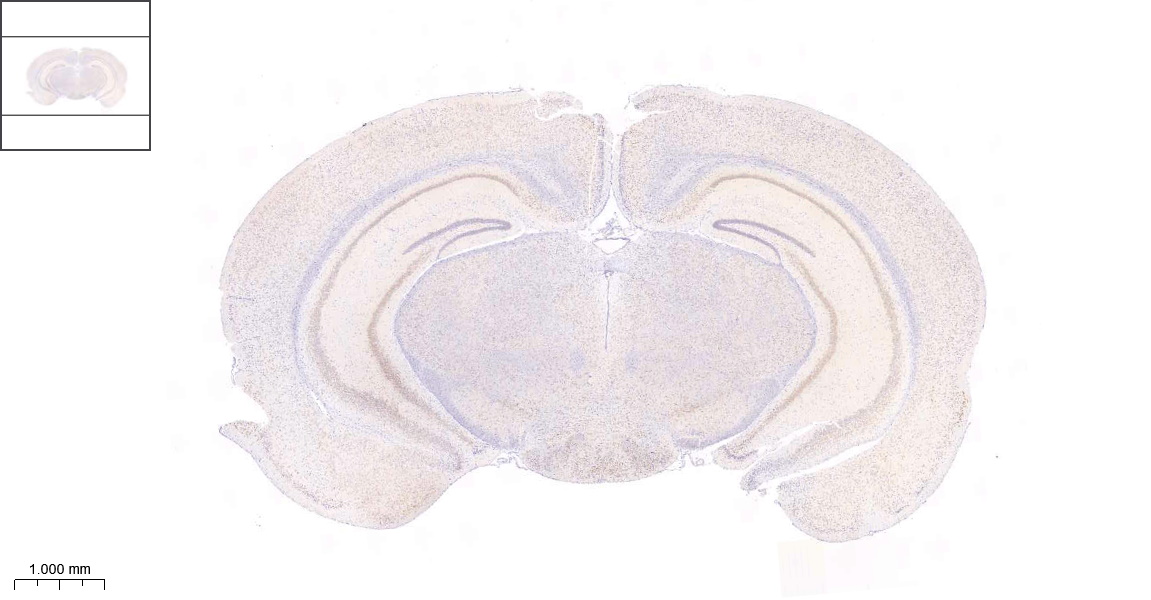


**AD 3 mon**


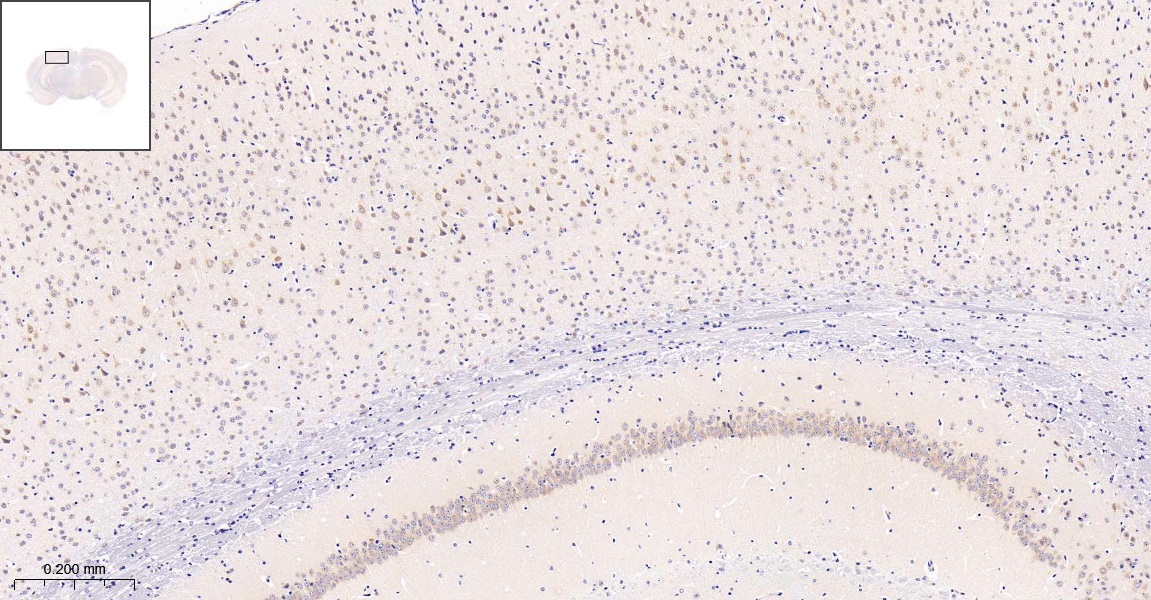

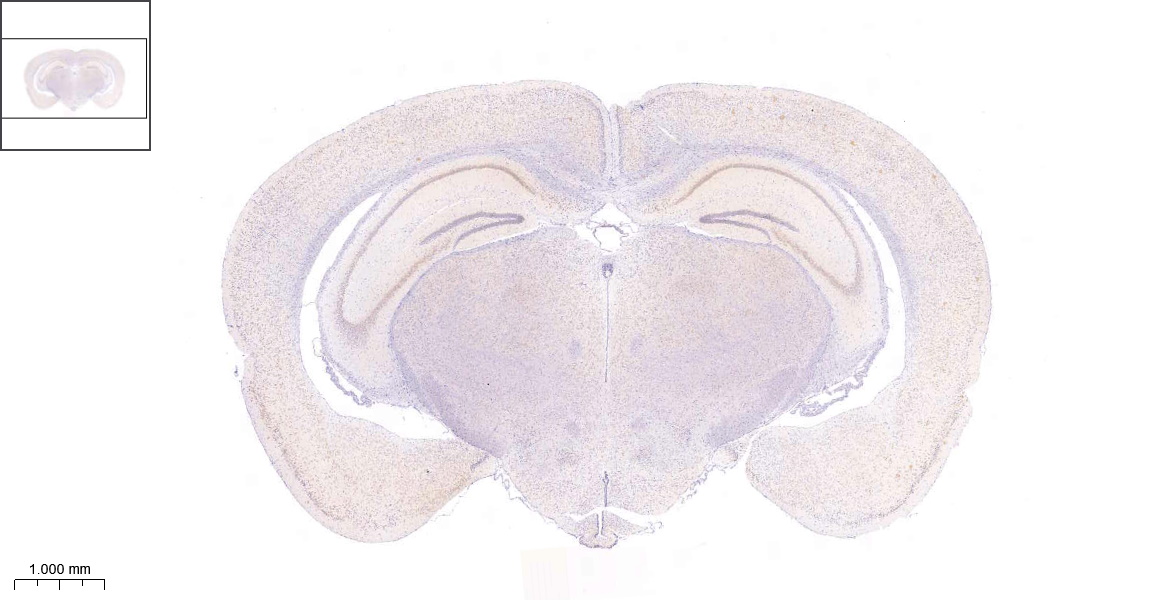


**AD 6 mon**


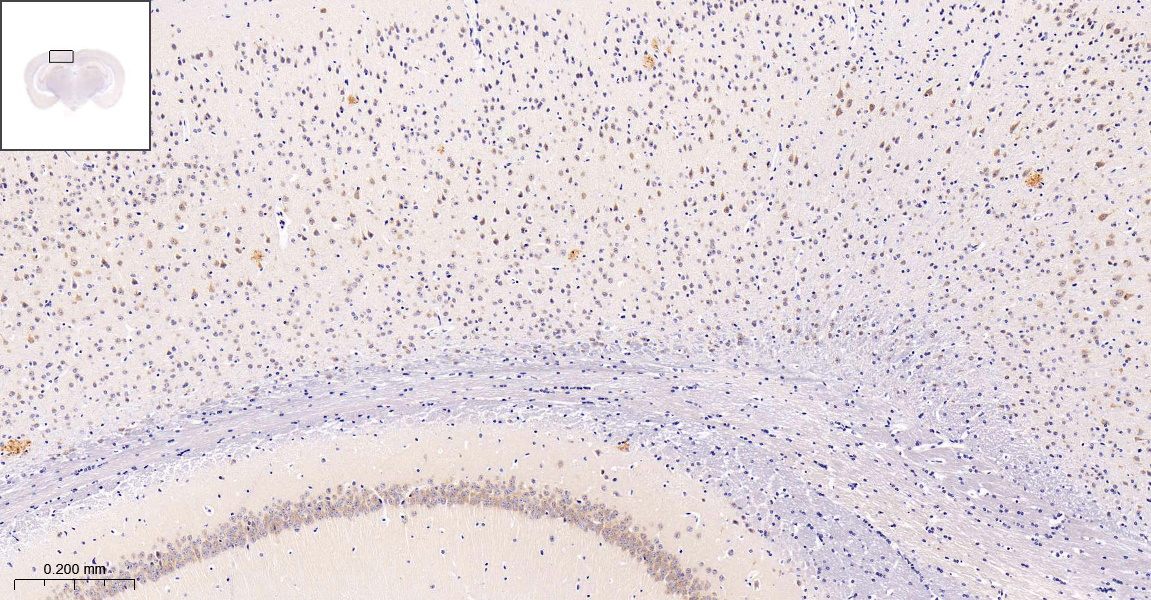


**1000 µm**

**200 µm**

**Figure. S9. H&E staining to evaluate the biosafety of A_40_-POs.**

Heart, liver, spleen, lung, kidney, and brain H&E staining of WT, AD, and A_40_-POs treated AD mice (14 days post-injection). (All scale bars = 50 μm, animals with the age of 12 months, n = 3)

**
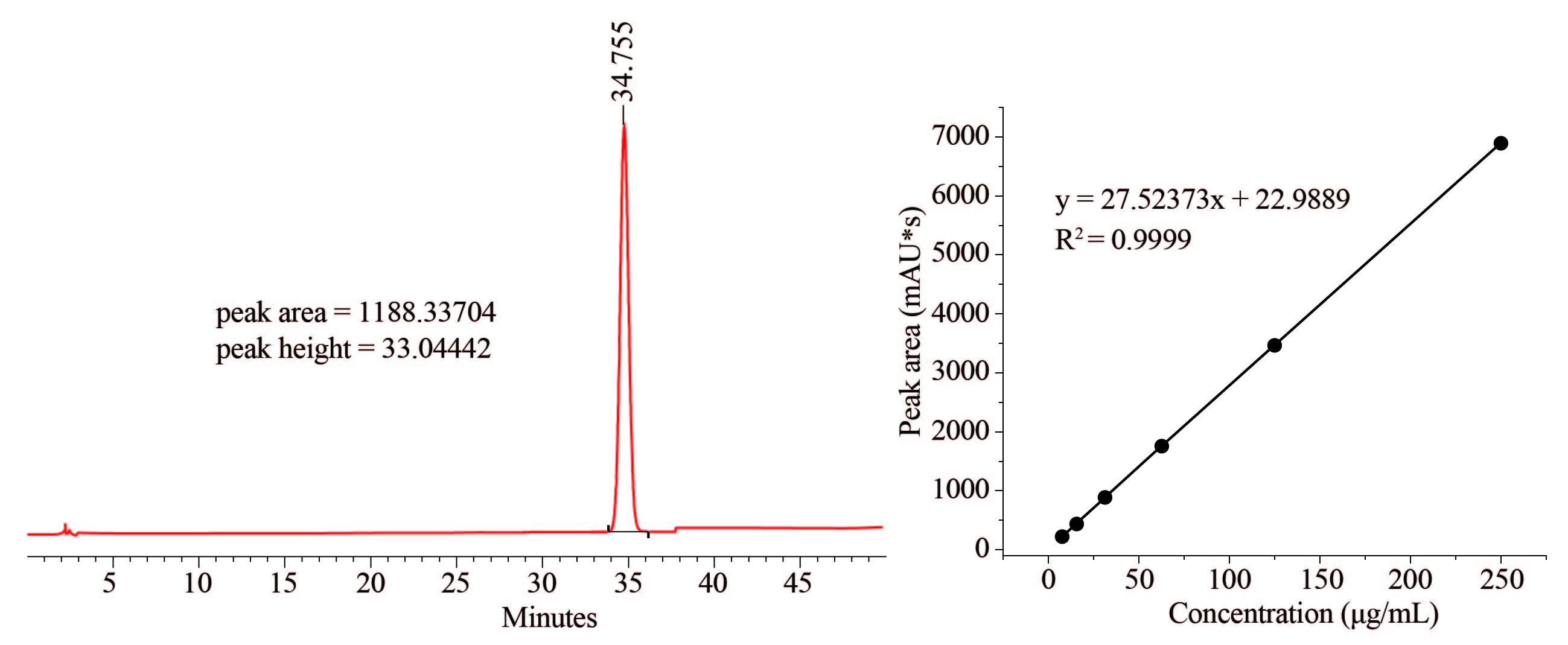
Figure. S10. HPLC methodology was employed to ascertain the encapsulation efficiency following the encapsulation of Donepezil within A_40_-POs.**

The peak area and peak height of the Donepezil@POs dialysate are depicted on the left. The corresponding standard curve is presented on the right. (Encapsulation efficiency = 20.68%).


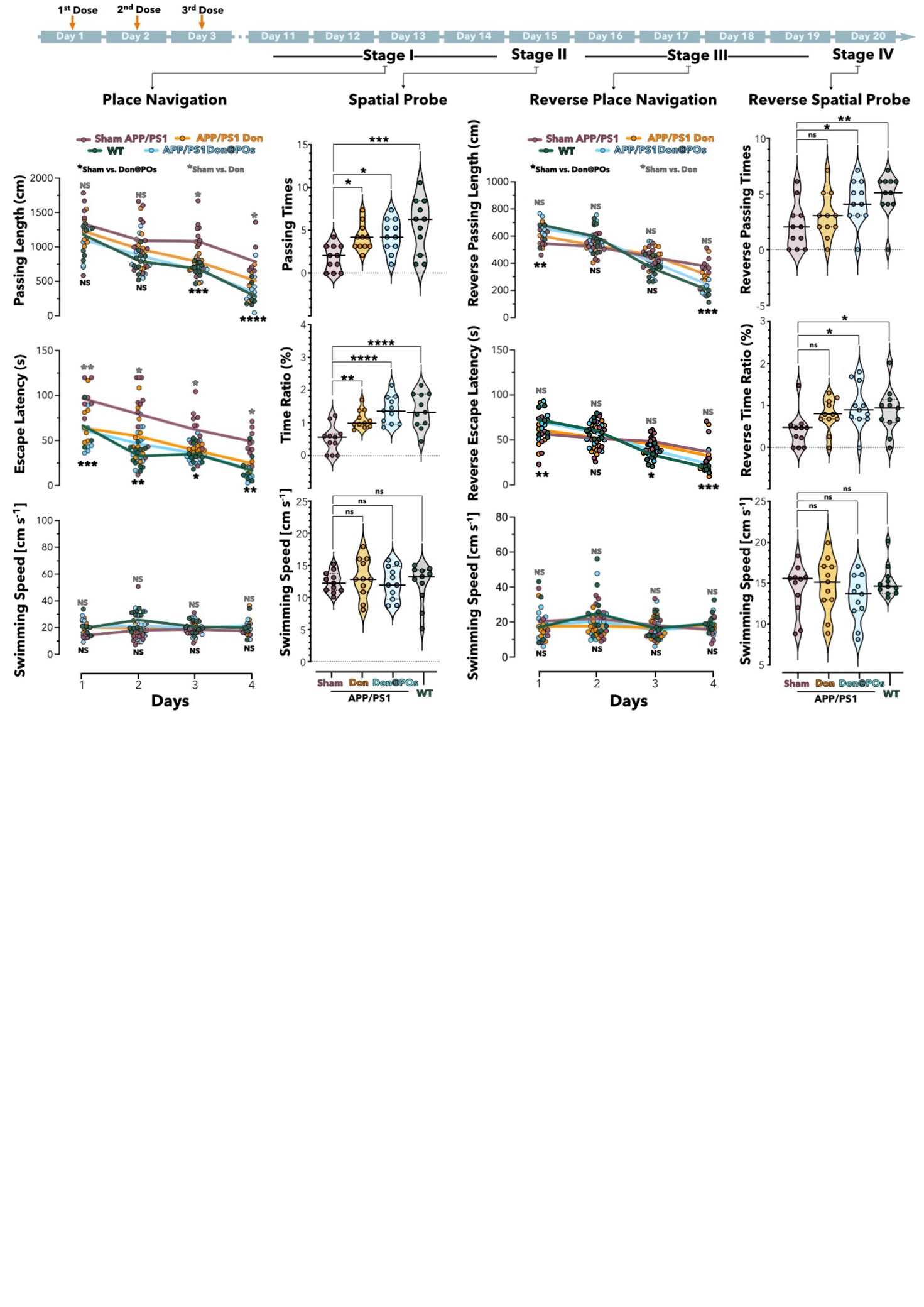
**Figure. S11. Donepezil@POs can treat AD and produce similar therapeutic effects to A_40_-POs, which are superior to the same dosage of free donepezil.**

Mice were injected with saline (Sham APP/PS1 and WT group), Donepezil@POs (Don@POs group, 10g/L 200μL) or Donepezil (Don group, 2g/lL 200μL) once per day for the first three mornings. Recovery was observed from days 4 to 10 under original rearing conditions. Place navigation (Stage I) test occurred on days 11-14, showing a gradual decrease in passing length and escape latency for finding the escape platform across all groups, with the APP/PS1 Don@POs group matching the WT level and significantly outperforming the Sham APP/PS1 group. Meanwhile, the Don group demonstrated space exploration capabilities superior to Sham APP/PS1. But not to the level of the Don@POs. Day 15th for the spatial probe (Stage II), APP/PS1 Don, APP/PS1 Don@POs, and WT groups demonstrated more passing times, and a higher percentage of time spent at the escape platform's original location. The reverse place navigation (Stage III) trial from days 16-19, with the platform moved to the opposite side (IV quadrant), the APP/PS1 Don@POs and WT groups initially took longer, indicating stronger spatial memory from the stage I and stage II. However, their reverse passing length and escape latency decreased rapidly over time and were significantly lower than those of the Sham APP/PS1 mice. In this stage, which is more difficult for mice compared to stage I and II, the Don group never showed a statistically significant difference in levels from the Sham APP/PS1 group. On day 20th, in the reverse spatial probe (Stage IV) test without the platform, Don@POs treated mice still outperformed the Sham APP/PS1 group. The Don group outperformed the Sham APP/PS1 group as well, but not to the level of the WT group. Place navigation trials (Stage I and III) were analysed using two-way ANOVA, while spatial probe trials (Stage II and IV) comparisons used one-way ANOVA. Significance levels are denoted as *p<0.05, **p<0.01, ***p<0.001, ****p<0.0001, with n ≥ 11).**
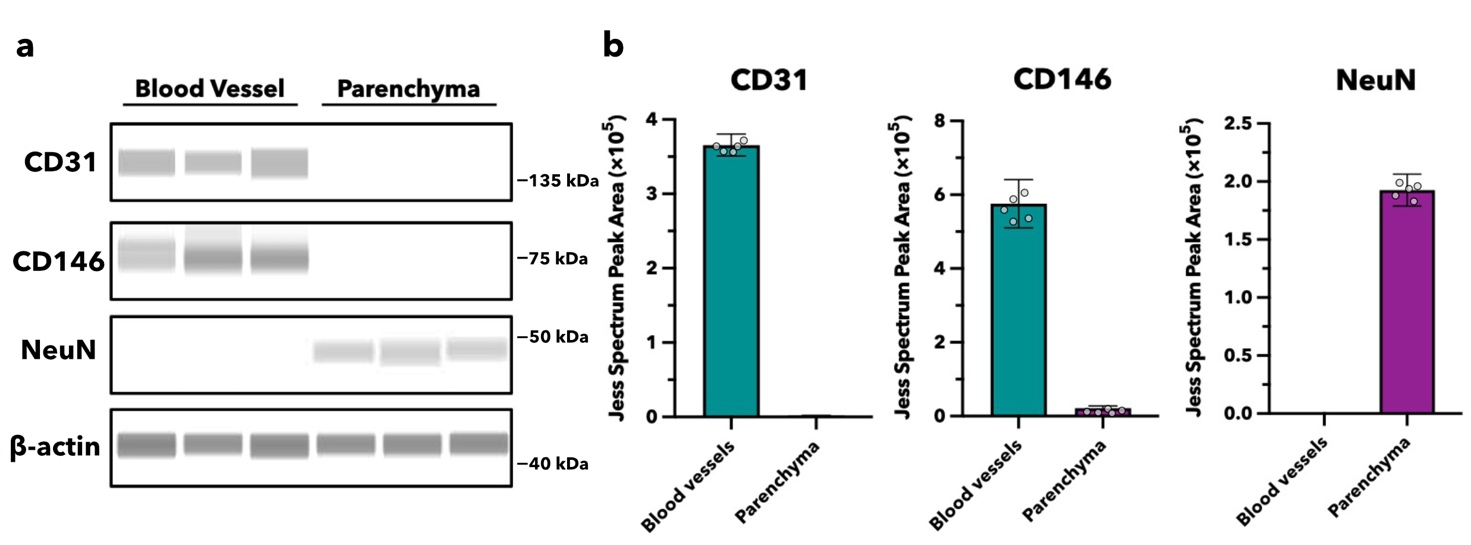
Figure. S12. The purity of the brain fractions extracted by the dextran-density-based protocol was detected by Digital Western Blot.**

Brain vessels and parenchyma separated by dextran were analyzed via Digital western blotting. The endothelial cell marker (CD31), pericyte marker (CD146), and neuronal cell marker (NeuN) in each brain fraction were detected individually. The band images (**a**) and the peak area of the grayscale values (**b**) were generated and calculated by the Jess system.

**
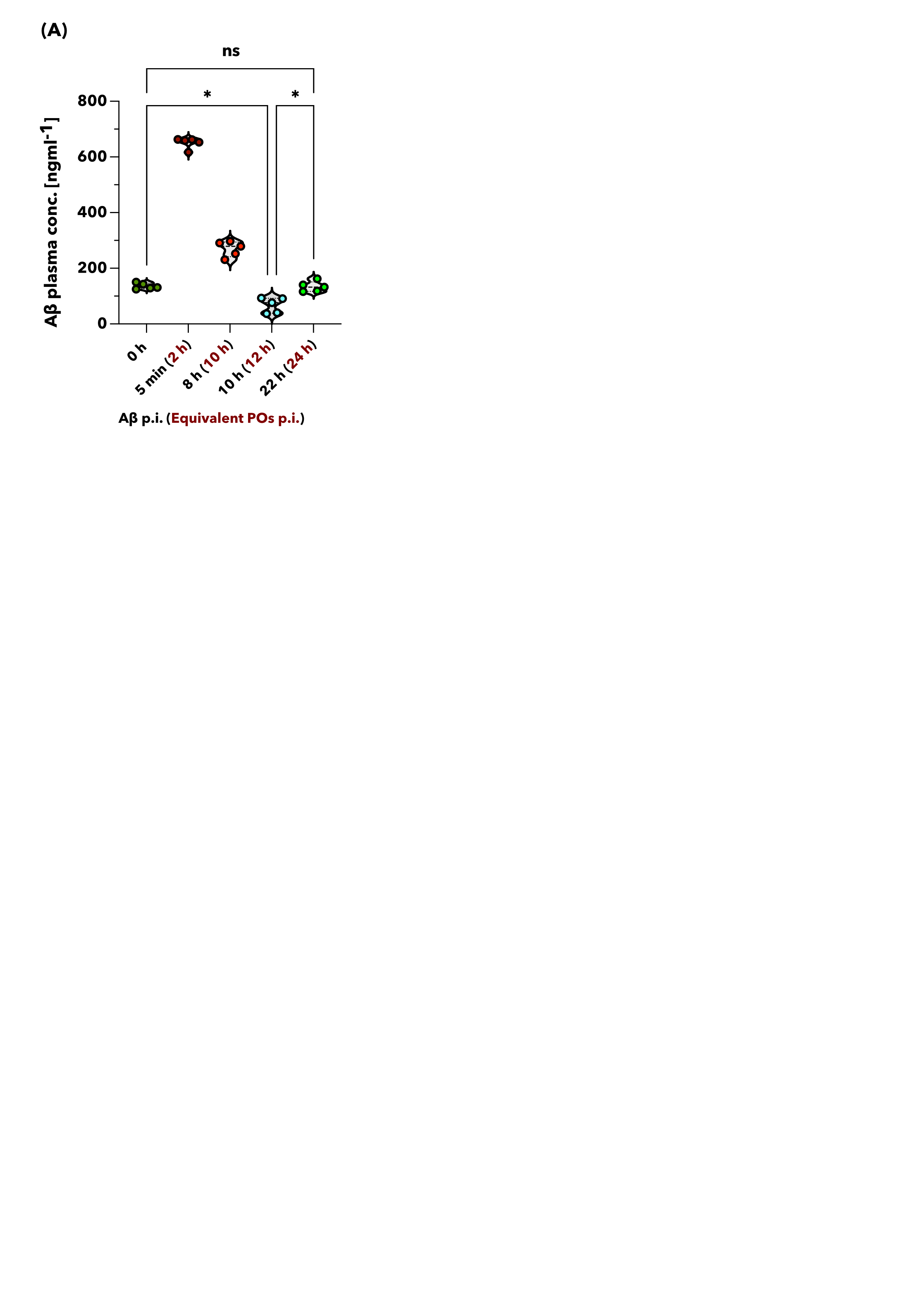
Figure. S13. The temporal evolution of blood Aβ concentration in mice following Aβ IV stimulation.**

Following the I.V. injection of Aβ into APP/PS1 mice, the plasma Aβ concentration was quantified at multiple time points using the ELISA method. The initial blood Aβ concentration post-injection was comparable to that observed 2 hours after A_40_-POs treatment, thereby simulating the temporal evolution of Aβ concentration in mouse blood following treatment. The x-axis is labeled as: Aβ time post-injection (equivalent to A_40_-POs time post-treatment).


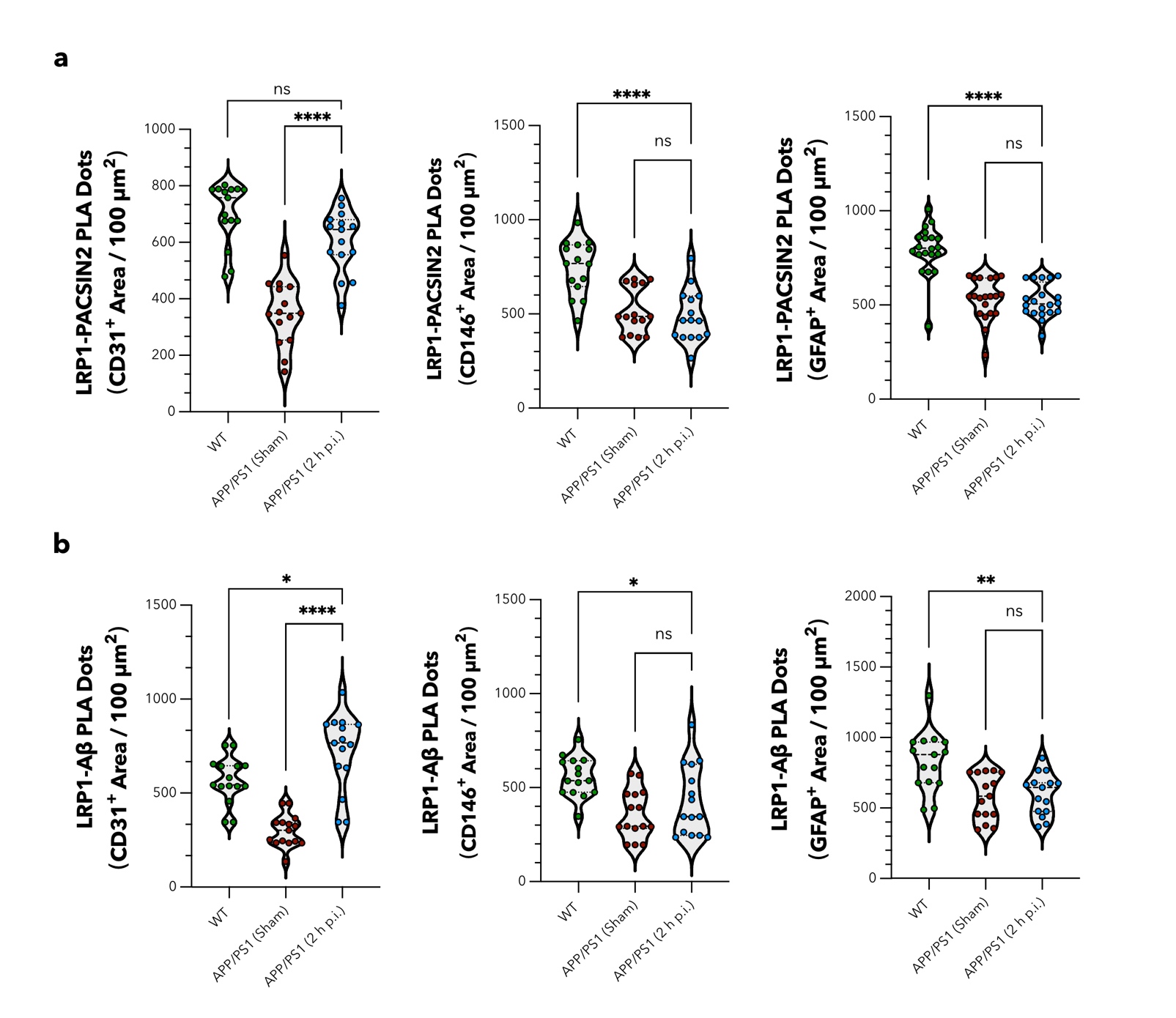
**Figure. S14. PLA-based detection of the synergistic interactions between LRP1 and other proteins in brain endothelial cells, astrocytes, and pericytes.**

The quantification of PLA signals for LRP1 and PACSIN2 was performed in the positive regions of endothelial cells (CD31), pericytes (CD146), and astrocytes (GFAP). Compared to the Sham group, A_40_-POs treatment resulted in upregulation of the LRP1-PACSIN2 pathway specifically in endothelial cells within the BBB (**a**). Additionally, the PLA signals for LRP1 and Aβ were quantified in the positive regions of endothelial cells (CD31), pericytes (CD146), and astrocytes (GFAP). Compared to the Sham group, A_40_-POs treatment led to an increase in LRP1-mediated Aβ transport exclusively in endothelial cells of the BBB (**b**). All PLA signals were normalized based on the area of positive regions for the cell markers (CD31, CD146, and GFAP), and the data are expressed as the number of signals per 100 μm².
